# Effect of chronic alcohol feeding using the Lieber-DeCarli diet on Alzheimer’s disease pathology in Tg2576 mice

**DOI:** 10.3389/fnagi.2025.1526571

**Published:** 2025-03-24

**Authors:** Devaraj V. Chandrashekar, Nataraj Jagadeesan, Tamara Abdullah, Rudy Chang, Ross A. Steinberg, Frankey Sanchez, Elias Khal, Joshua Yang, David H. Cribbs, Derick Han, Rachita K. Sumbria

**Affiliations:** ^1^Department of Biomedical and Pharmaceutical Sciences, School of Pharmacy, Chapman University, Irvine, CA, United States; ^2^School of Pharmacy and Health Sciences, Keck Graduate Institute, Claremont, CA, United States; ^3^Institute for Memory Impairments and Neurological Disorders, University of California, Irvine, Irvine, CA, United States; ^4^Department of Neurology, University of California, Irvine, Irvine, CA, United States

**Keywords:** alcohol, Aβ, Tg2576, LRP-1, Alzheimer’s disease, Lieber-DeCarli, liver

## Abstract

**Background:**

Chronic alcohol drinking is a modifiable risk factor for Alzheimer’s disease (AD), but underlying mechanisms remain poorly understood. Most studies of alcohol feeding to AD mice have utilized young mice and delivered alcohol in drinking water without controlling nutritional intake.

**Objective:**

To study the impact of Lieber-DeCarli (LDC) liquid alcohol diet, which balances nutritional intake, on AD pathology of aged Tg2576 and wild-type (WT) mice, which is unexplored.

**Methods:**

13-month-old male and female Tg2576 or WT mice were fed LDC diet (5% ethanol or control) for six weeks (*n* = 11-13/group). Exploration (open-field test) and spatial reference memory (Y-maze test) were assessed after six weeks, and brains and livers were studied for Aβ levels, and Aβ synthesis and transport proteins (APP and LRP-1). Neuroinflammation, blood–brain barrier function, and synaptic health were studied using immunoassays.

**Results:**

LDC alcohol feeding significantly reduced survival (*p* < 0.05) and spatial memory (*p* < 0.05) in Tg2576 mice, but not in WT mice. Alcohol feeding increased (*p* < 0.001) insoluble endogenous mouse Aβ_1-42_ and reduced microgliosis (*p* < 0.05) in Tg2576 mice brains, but not in WT mice. LDC alcohol feeding to Tg2576 mice caused mild liver injury, and important amyloidosis-relevant hepatic proteins (LRP-1 and APP) were largely unaltered. However, brain Aβ and microgliosis were positively correlated (*p* < 0.05) with serum alanine aminotransferase, a marker of liver injury, in Tg2576 mice.

**Conclusion:**

Chronic alcohol intake, resulting in mild liver injury, caused modest but significant AD-relevant changes in aged Tg2576 mice, which correlated with liver injury; the latter suggests significant liver-brain crosstalk in an AD model of moderate alcohol intake.

## 1 Introduction

Alzheimer’s disease (AD) is a progressive neurodegenerative disorder characterized by the accumulation of amyloid-*β* (Aβ) plaques, formation of intracellular neurofibrillary tangles (NFTs), neuronal excitotoxicity, and neuronal apoptosis (Alzheimer's Association, 2022). Epidemiological studies suggest that heavy alcohol intake associated with alcohol use disorder (AUD) is a major risk factor for the development and progression of AD. Heavy alcohol consumption (defined as >14 drink units/week) has been shown to promote brain atrophy, alter brain structure, and promote cognitive impairment (Mechtcheriakov et al., 2007; Heymann et al., 2016; Jeon et al., 2023). While most epidemiological studies suggest that heavy alcohol drinking aggravates AD progression, there are few studies suggesting that low alcohol intake may protect against AD (Beydoun et al., 2014; Chan et al., 2010). However, the potential of low alcohol intake to protect against AD is not universally observed and highly controversial.

Alcohol feeding studies in rodent models, especially in AD transgenic mice, have generally supported the notion that chronic alcohol intake promotes AD pathology, including alterations in microglial response, decline in cognitive tests, and increase in Aβ, amyloid precursor protein (APP) and other AD-relevant biomarkers. In 3xTg-AD mice (3-month-old, male and female), alcohol feeding (25% w/v + saccharin 0.1% w/v) in drinking water for four months impaired spatial memory, and increased Aβ (42/40) ratio, total tau, and phosphorylated tau in the brain (Hoffman et al., 2019). Similarly, in APP23/PS45 mice (2-month-old, sex not mentioned), alcohol feeding (20% v/v + 0.07% w/v saccharin) in drinking water for four weeks increased learning and memory impairment and brain levels of Aβ40, Aβ42, APP, and beta-site amyloid precursor protein cleaving enzyme 1 (BACE-1) (Huang et al., 2018). Further, in APP/PS1 mice (5.5-month-old, male), alcohol feeding (20% v/v) in drinking water for ten weeks during the dark cycle increased locomotor activity and hippocampal Aβ40 (Day et al., 2023). Apart from models using alcohol in drinking water, chronic binge alcohol oral dosing at 10 mL/kg for two months to APPswe/PS1dE9 mice (4-month-old, male) increased blood–brain barrier (BBB) disruption and aggravated cognitive decline (Wei et al., 2022). Similarly, oral adolescent intermittent ethanol dosing at a dose of 5 g/kg (25% w/v in water) increased cognitive decline and Aβ and tau pathology in adult female 3xTg-AD mice (Barnett et al., 2022). These studies have been detailed in a review examining the pathogenic effects of different alcohol-feeding regimens on different AD models (Chandrashekar et al., 2023).

Although many studies have been performed feeding alcohol to AD transgenic mice, several gaps in knowledge still exist. First, most studies have utilized alcohol delivery by mixing alcohol in drinking water or delivery using daily gavage or injection. The delivery of chronic alcohol to animals is difficult due to their natural aversion to alcohol, and no model can mimic liver pathology seen in heavy drinkers with AUD. All models of alcohol delivery to rodents have strengths and weaknesses. The drawback of most studies that delivered alcohol by mixing in drinking water or daily gavage/injection is the lack of nutritional intake control. Because high calories are associated with alcohol intake, animals given alcohol in drinking water or gavage/injected will eat less, and thus obtain different nutrients from their diet. Since AD is associated with metabolic dysregulation (Yan et al., 2020), having different nutritional intake using these methods of alcohol delivery may create metabolic discrepancy. Alcohol feeding using a liquid diet (i.e., Lieber-DeCarli (LDC) liquid diet) or through intragastric feeding can better balance nutritional intake through pair feeding (Bertola et al., 2013; Tsukamoto et al., 1985). Second, most studies examining the effects of alcohol on AD mice have primarily focused on measuring human Aβ levels without focusing on mouse APP or Aβ since the widely used AD transgenic mice, such as Tg2576 mice and APP/PS1 mice, overexpress mutant human APP in the brain (Sasaguri et al., 2017; Sturchler-Pierrat et al., 1997). To obtain a holistic understanding of how alcohol may promote AD in transgenic mice, both endogenous mouse APP and overexpressed human APP should be measured along with both human and mouse Aβ levels. Third, most studies examining alcohol’s effect on AD mice have generally utilized younger transgenic mice (2–8-month-old) (Chandrashekar et al., 2023). Alcohol feeding in young mice is much more common, as younger mice tolerate alcohol better than older mice. Thus, while it appears that alcohol feeding to younger AD mice accelerates some aspects of AD pathology, the question of whether chronic alcohol feeding to older AD mice will similarly accelerate AD pathology has not been extensively explored. This is important because age is the biggest known risk factor for AD dementia (Alzheimer's Association, 2022). Further alcohol consumption generally remains high in patients with dementia, with an estimated 25% of patients diagnosed with dementia also having AUD (Caputo et al., 2012). In addition, it has been reported that alcohol consumption in patients with early AD accelerates cognitive decline and AD progression (Heymann et al., 2016). Thus, more alcohol feeding studies are needed in older AD mice with AD pathology that is not full-blown to gain greater mechanistic insights into how alcohol affects the progression of AD in its early stages. Finally, the impact of moderate chronic alcohol-induced hepatic changes on AD pathology has been understudied in alcohol-feeding studies in AD transgenic mice. Our recent studies show that chronic intragastric alcohol feeding, a method that uses a catheter to continuously deliver the highest levels of alcohol to mimic heavy alcohol consumption, to C57BL/6 J and APP/PS1 mice, results in AD-relevant brain changes that are associated with hepatic Aβ dysregulation (Garcia et al., 2022; Chandrashekar et al., 2024).

To fill these gaps in knowledge, in this study, we examined the effect of chronic moderate alcohol feeding using the LDC liquid diet on AD pathology in aged (13-month-old) male and female Tg2576 AD mice. By this age, Tg2576 mice show diffuse Aβ pathology which continues to advance with age, and robust Aβ pathology similar to human AD brains, is seen in older Tg2576 mice (Kawarabayashi et al., 2001). The LDC allows for nutritional balance during chronic alcohol feeding and is a model of moderate alcohol drinking based on liver pathology (Brandon-Warner et al., 2012). This study measured both human and mouse APP expression and Aβ levels to obtain a holistic view of changes in total Aβ levels with chronic moderate alcohol feeding in aged Tg2576 mice. In addition, to study the liver-brain axis in the development of AD pathology (Chandrashekar et al., 2023), we explored the association between hepatic changes caused by chronic moderate alcohol feeding and AD pathology.

## 2 Materials and methods

### 2.1 Animals and experimental design

**Figure 1:**
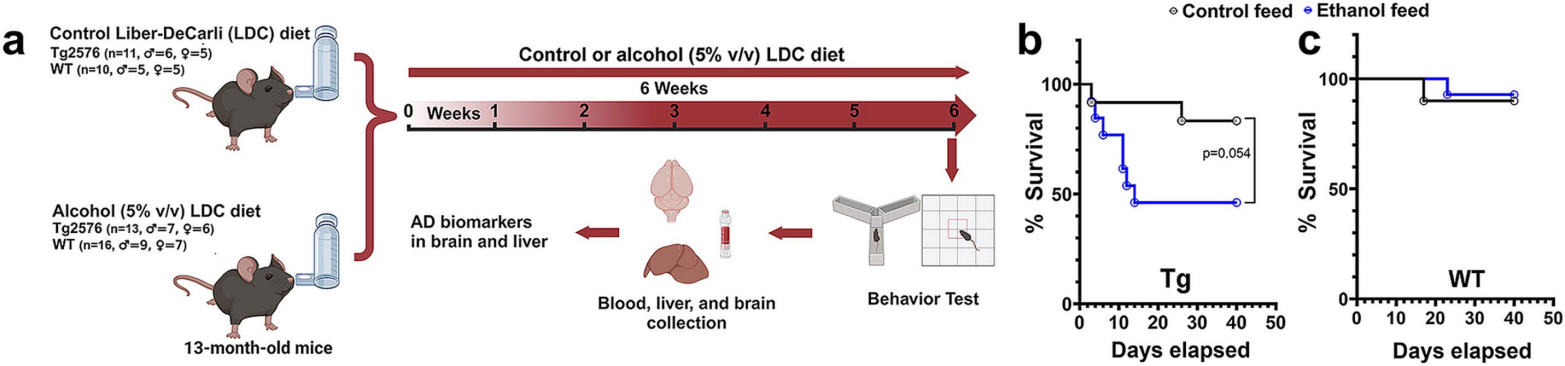
Effect of alcohol-LDC feeding on survival in the Tg2576 mice and wild-type (WT)-littermates. Schematic of the experimental design **(a)**. Survival curves show a lower survival for chronic alcohol-LDC-diet-fed Tg2576 mice compared to the control-diet-fed Tg2576 mice (*p* = 0.054) **(b)**. Survival curves show no significant change in survival between the chronic alcohol-LDC-diet-fed and control-diet-fed WT mice **(c)**. The survival curves were compared using the Log-rank test.

A total of 50 male and female mice were used: Tg2576 (B6; SJL-Tg (APPSWE) 2576Kha) (*n* = 24, 13 males and 11 females) and wild-type (WT) littermate (*n* = 26, 14 males, 12 females) mice were bred and provided by Dr. David Cribbs from University of California, Irvine (UCI), and were aged at the Chapman University vivarium for the experiments. The Tg2576 mice were on the B6;SJL mixed background. All the animals were housed in a temperature and humidity-controlled animal care facility under a 12-h light–dark cycle, with free access to food ad libitum. The alcohol-feeding experiments were approved by the Chapman University Institutional Animal Care and Use Committee (protocol number 2020-1170). Thirteen-month-old male (*n* = 5-9/group) and female (*n* = 5-7/group) Tg2576 and WT mice were randomized according to their body weights and age into two groups, the control group (fed with control liquid diet) and the alcohol group (fed with alcohol liquid diet) (Figure 1a). The female mice were housed in pairs, while the male mice were housed individually because of their territorial nature that can cause significant aggression in male mice.

### 2.2 LDC diet preparation

The control and alcohol liquid diets were prepared fresh every three days according to the method described previously (Bertola et al., 2013). Briefly, the control liquid diet was prepared by thoroughly mixing 225 g of dry mix (F1259SP, Bio-Serve, Flemington, NJ, USA) with ~860 mL of drinking water. The prepared feed was stored at 4°C and used up within three days after preparation. The alcohol liquid diet (5% v/v, ethanol) was prepared by mixing 133 g of the dry mix (F1258SP, Bio-Serve, Flemington, NJ, USA), 20.5 g of maltose dextrin with 910 mL of drinking water. Just before feeding, 52.5 mL of ethanol (95%) was added for every 1 liter of the feed, followed by blending and animal feeding.

### 2.3 Feeding regimen

All the animals [(control: *n* = 11 (5 females and 6 males) for Tg2576 mice, and *n* = 10 (5 females and 5 males) for WT mice) or (alcohol: *n* = 13 (6 females and 7 males) for Tg2576 mice, and *n* = 16 (7 females and 9 males) for WT mice)] were acclimatized to the tube feeding method by feeding them with the control LDC diet for five days. For this, ~30 mL (for a single mouse) or 50 mL (for paired mice) of the LDC diet was filled in the glass feeding tubes, capped tightly, weighed, and placed in the cages using tube holders. The tubes were weighed to record the feed consumption after 24 h, followed by washing and refilling the tubes with the fresh LDC control diet daily for the next four days. No additional drinking water or diet was given to these animals. After five days of acclimatization, the alcohol group received the alcohol LDC diet containing 5% (v/v) alcohol for the next six weeks. For this, ~25 mL (for a single mouse) or 40 mL (for paired mice) of the alcohol LDC diet was fed, and accordingly, the control LDC diet (~15 mL (for a single mouse) or 25 mL (for paired mice) was fed to the control group (Bertola et al., 2013). The nutritional/caloric intake of alcohol-fed and control-fed mice were matched by pair feeding, which involved: 1) recording feed consumption daily per mouse, and 2) adjusting the volume of the control LDC diet according to the average feed intake per mouse of the alcohol LDC diet group (Bertola et al., 2013). The time of feeding was kept constant (between 3.00 pm to 5.00 pm). The body weights of the animals were monitored daily.

After six weeks of control (*n* = 9, male = 6, female = 3 for Tg2576 mice and *n* = 9, male = 4, female = 5 for WT mice) or alcohol (*n* = 6, male = 4, female = 2 for Tg2576 mice, and *n* = 13, male = 6, female = 7 for WT mice) LDC diet, the animals were subjected to the open-field and Y-maze tests to assess their exploratory behavior and spatial reference memory, respectively. On the following day, terminal weights were measured, blood was collected for serum/plasma analysis, and the animals were euthanized with a lethal dose of Euthasol (150 mg/kg, intraperitoneal), followed by cardiac perfusion with ice-cold phosphate-buffered saline (PBS) and organ harvesting (brain and liver). The right hemi-brains were fixed in 4% paraformaldehyde (PFA) for immunostaining, and the left hemi-brains were snap frozen in liquid nitrogen for Western blotting and ELISA.

### 2.4 Blood alcohol levels

Blood alcohol levels were monitored at five hours post-feed placement following the last dose of the feed. For this, the plasma sample was diluted with 10x ethanol assay buffer and analyzed for alcohol concentration using a commercially available ethanol assay kit (#ab65343, Abcam, Waltham, MA, USA) as per the vendor’s instructions. Briefly, 50 μL of standard/sample was mixed with the same volume of ethanol assay kit reaction mixture and incubated at 37°C for 30 min. The absorbance was read at 570 nm using a UV–visible spectrophotometer, and the final blood alcohol concentrations were calculated in mg/dL (Zehendner et al., 2013).

### 2.5 Open field test

The open-field test was done in two phases, (i) at the start of the experiment (baseline recordings), and (ii) at six weeks after the alcohol/control LDC diet (post-treatment recordings) to assess the exploratory and anxiety-like behavior of the animals (Jagadeesan et al., 2024). The test used a square-shaped white open-field box (72 cm x 72 cm with 36 cm walls) with a center square (36 cm x 36 cm). Briefly, the mouse was placed in the open-field box to record their movements for 5 min. The mean speed and distance traveled by the mouse were evaluated using the SMART Video Tracking Software (Panlab, Harvard Apparatus, MA, USA) (Ou et al., 2022). The final data were normalized to the baseline values. The tendency to explore the center zone was used to assess anxiety-like behavior.

### 2.6 Y-maze test

To evaluate the spatial reference memory, a Y-maze apparatus consisting of three equal radial arms (30 × 5 cm, start arm, novel arm, and familiar arm), placed at the ground level, was used. The study was performed in two phases, the training phase and the testing phase (Ou et al., 2022). For the training phase, the novel arm was blocked, and the mouse was allowed to explore the start arm and familiar arm for 8 min by placing the animal in the start arm. After 30 min, the blocked novel arm was opened, and the mouse was allowed to explore all three arms for an additional 8 min. Entries in the novel arm and latency to enter the novel arm were measured using the SMART Video Tracking Software (Panlab, Harvard Apparatus, MA, USA).

### 2.7 Liver injury analysis

Terminal serum was used for the measurement of the following liver injury biomarkers: alanine aminotransferase (ALT), alkaline phosphatase (ALP), albumin, blood urea nitrogen (BUN), and cholesterol by an automated analyzer (Abaxis Vetscan-VS2, Zoetis, NJ, USA) using the mammalian liver profile rotors. Briefly, serum samples were diluted (1:1) with normal saline, and ~ 100 μL of sample was loaded onto the rotor and analyzed.

### 2.8 Brain cryosectioning

The right hemi-brains were initially fixed with 4% PFA in PBS for 24 h, and then cryoprotected with 10, 20, and 30% sucrose solution at 4°C over a period of 24 h each. Samples were frozen using powdered dry ice and stored at −80°C until further analysis. The fixed-frozen hemi-brains were mounted in a freezing cryostat (Micron Instruments, CA, USA) using the Tissue-Tek OCT compound (Fisher Scientific, MA, USA), and sectioned into 20 μm sagittal sections. For each mouse, three sections (600 μm apart) were used for immunostaining (Ou et al., 2022).

### 2.9 6E10 immunostaining and quantification

Alexa Fluor 488-labeled monoclonal antibody (Bio Legend, CA, USA) against human Aβ (1–16) was used to stain Aβ deposits in the brain sections. Briefly, free-floating sagittal brain sections were washed in PBS followed by antigen retrieval with 70% formic acid. After washing in distilled water, sections were blocked with 0.5% bovine serum albumin (BSA) in PBS containing 0.3% TritonX-100 for 60 min at room temperature (RT). Sections were then stained with the 6E10 primary antibody solution (1:1000) overnight at 4°C. After washing, brains sections were mounted onto glass slides and cover slipped using Vectamount aqueous mounting media (Vector Laboratories, CA, USA). The slides were imaged using a BZ-X710 Keyence fluorescence microscope (Keyence, IL, USA), and the total Aβ stain area, counts, and size were quantified using the NIH ImageJ software (Bethesda, MD, USA) by two observers blinded to the treatment groups.

### 2.10 Iba-1 immunostaining and quantification

Free-floating sagittal brain sections were washed in PBS followed by incubating with the blocking buffer (0.5% BSA in PBS containing 0.3% Triton X-100) for 60 min at RT. Sections were incubated overnight at 4°C with the anti-Iba-1 polyclonal rabbit antibody solution ((1:1000); Wako, VA, USA) prepared in the blocking buffer. After washing, tissue sections were incubated in the dark with the Alexa Fluor 488 donkey anti-rabbit IgG (1:1000; Bio Legend; CA, USA) in blocking buffer for 2 h at RT. Sections were mounted on the glass slides and coverslipped using Vectamount aqueous mounting media (Vector Laboratories, CA, USA). Three regions in the cortex and two regions in the hippocampus were imaged at 40x using a Nikon ECLIPSE Ti2 Confocal Microscope (Nikon Instruments Inc., NY, USA), and the microglial positive area and the total microglia count for each image were quantified using the NIH ImageJ software (Bethesda, MD, USA). The total number of microglia per mouse was the sum of the total microglia in each image. Microglia were further categorized into ramified, activated, and dystrophic (Paasila et al., 2019) based on their morphology and counted using NIH ImageJ. All the quantification was done by three readers blinded to the treatment groups.

### 2.11 Brain homogenization

The frozen left hemi brains without the cerebellum were pulverized uniformly on dry ice and homogenized with ten volumes of tris-buffered saline (TBS) buffer (50 mM Tris–HCl, pH 7.6, 150 mM NaCl, 5 mM EDTA, 2-mM 1,10-phenanthroline with Roche complete EDTA-free Mini protease inhibitor). The homogenate was centrifuged at 100,000 g for 1 h at 4°C, and the supernatant (TBS fractions) was collected into multiple aliquots and stored at −80°C until further analysis. The pellet from the above step was resuspended in 10 volumes of homogenizing buffer (5 M guanidine HCl (Gu-HCl), 0.05 M Tris, pH 8.0), homogenized, and allowed to shake on a rotor for 2 h at RT. The homogenate was centrifuged at 20,800 g for 15 min at RT, and the supernatant (Gu-HCl fractions) was collected and stored at −80°C until further analysis. Similarly, the pulverized brain samples were homogenized with 15 volumes of radioimmunoprecipitation assay buffer (RIPA) with Roche complete EDTA-free Mini protease inhibitor and allowed to shake on a rotor for 1 h at 4°C. The homogenate was centrifuged at 12,000 g for 20 min at 4°C, and the supernatant (RIPA fractions) was collected and stored at −80°C until further analysis. The total protein concentrations of the TBS, Gu-HCl, and RIPA brain fractions were determined by the bicinchoninic acid (BCA) method (Pierce Chemical Co., Rockford, IL, USA).

### 2.12 Quantification of human and mouse Aβ_1-42_ by ELISA

TBS-soluble and Gu-HCl-soluble brain fractions, and plasma samples were used to quantify Aβ_1-42_ using commercially available human (#KHB3441, Thermo-Fisher Scientific, CA, USA) and mouse (#KMB3441, Thermo-Fisher Scientific, CA, USA) Aβ_1-42_ ELISA Kits, as per the vendor instructions.

### 2.13 Liver histology

Livers were removed, fixed with 10% buffered formalin, embedded in paraffin, and cut into 5-μm thick sections. A subset of tissues (*n* = 4 per group) was stained with hematoxylin/eosin (*Η*&*Ε*) by the Pathology Core at UC Irvine. Liver steatosis scoring was performed by a reader blinded to the treatment group using a scale of 0–3 as follows: steatosis <5% of the image = score 0, steatosis between 5–33% of the image = score 1, steatosis between 34–66% of the image = score 2, and steatosis ≥66% of the image = score 3 (Lackner et al., 2021).

### 2.14 Real-time quantitative PCR

LRP-1, APP, and actin mRNA levels in the liver were assessed by RT-PCR. RNA was extracted from liver following homogenization using column-based isolation (PureLink RNA Mini Kit, Invitrogen). RNA was quantified using Tecan NanoQuant Infinite 200 Pro and matched to a concentration of 50 ng of RNA. cDNA was then reverse transcribed using Invitrogen SuperScript III First-Strand Synthesis SuperMix (utilizing Oligo(dT)) and thermocycler. cDNA was subsequently diluted to 1:10 with RNase-free water. For RT-PCR, 2 μL of this dilution was used per well in conjunction with 12.5 μL SYBR Green (Applied Biosystems PowerSYBR Green PCR Master Mix), 9.5 μL RNase-free water, and 1 μL of mixed forward and reverse primer (Integrated DNA Technologies - Coralville, Iowa) at a concentration of 10 μM, for a total of 25 μL per well. Non-template controls were used to test for primer contamination and/or primer-dimer interactions. To assess primer validity, a standard curve was conducted to ensure efficiency percentages were between 90–110%. Samples were loaded into a 96 well plate with each condition run in triplicate. A Roche Light cycler 96 was set for a preincubation cycle of 10 min at 95°C, followed by 40 cycles of 2-step amplification at 95°C for 15 s and 60°C for 60 s, and finally a melting curve of 95°C for 15 s, 65°C for 15 s, and a ramp toward 95°C increasing at a speed of 0.2°C per second. The resulting Cq data was collected from Roche’s Light cycler 96 SW 1.1 software and was averaged and analyzed using the 2^-ΔΔCT^ method. In order to assess differences resulting from ethanol consumption on LRP-1 and APP transcription, we reverse transcribed RNA isolated from liver homogenate and conducted RT-PCR on the resultant cDNA. *β*-actin was chosen as control as we previously described (Garcia et al., 2022).

### 2.15 Western blot analysis

The protein expression of the AD biomarkers in the TBS-soluble and RIPA brain fractions was determined by Western blot analysis. In brief, the TBS-soluble and RIPA brain fraction samples (20–100 μg protein/lane) processed in 4x Laemmli buffer with 10% *β*-mercaptoethanol were separated by SDS-polyacrylamide gel electrophoresis on a precast 4–20% MP TGX gel (Bio-Rad, Hercules, CA, USA) at a constant voltage (100 V) for 60 min. Proteins were transferred with the Bio-Rad Trans-Blot Turbo Transfer System (Bio-Rad, Hercules, CA, USA) onto 0.45 μm polyvinylidene fluoride (PVDF) membranes. The PVDF membranes were blocked with 5% milk for one hour at RT. The blots were then incubated overnight at 4°C with primary antibodies diluted in 3% milk in 1x TBS for brain analysis: human APP (1:1000, #803001, Bio-Legend, San Diego, CA, USA), ZO-1 (1:1000, #40-2200, Bio-Legend, San Diego, CA, USA), LRP-1 (1:1000, #64099, Cell Signaling, Danvers, MA, USA), PSD-95 (1:1000, #sc-32290, Santa Cruz, CA, USA), claudin-5 (1:1000, #sc-374221, Santa Cruz, CA, USA), presenilin 1 (1:1000, #5643, Cell Signaling, Danvers, MA, USA), or *β*-actin (loading control, 1:1000, #sc-47778, Santa Cruz, CA, USA). For the liver, samples were homogenized in RIPA buffer (Thermo Fisher Scientific, Waltham, MA, USA) containing protease/phosphatase inhibitor cocktail (Cell Signaling, Danvers, MA, USA). Protein electrophoresis occurred on 8–12% SDS polyacrylamide gels (Bio-Rad, Hercules, CA, USA). Subsequently, proteins were transferred to nitrocellulose or PVDF membranes and blots were blocked with 5% (w/v) nonfat milk dissolved in TBS with Tween-20. Antibodies to LRP-1 (#64099), APP (#76600), and *β*-actin (#3700) were obtained from Cell Signaling (Danvers, MA, USA). After washing, the blots were incubated with HRP-conjugated IgG anti-rabbit (1:1000, #7074, Cell Signaling, Danvers, MA, USA), or HRP-conjugated anti-mouse IgG kappa (1:1000, #sc-516102, Santa Cruz, CA, USA) secondary antibodies for one hour at RT followed by washing and visualization of bands with the Bio-Rad Chemi Doc Imager system (Bio-Rad, Hercules, CA, USA) after addition of enhanced chemiluminescence (ECL) reagent. Image Lab software (version 6.1, Bio-Rad, Hercules, CA, USA) was used to quantify the intensity of the Western blot bands, and protein expression was normalized to the β-actin protein expression.

### 2.16 Statistical analysis

All data are expressed as mean ± SEM, and the statistical analysis was performed with GraphPad Prism (v9.04, La Jolla, CA, USA). Sex differences were not studied due to low number of female mice in the Tg2576 group, and male and female data were combined. No overt sex-specific effects were observed for the mice that survived except for the Iba-1 analysis, where effects were observed only in the male mice. Grubb’s test was used to identify outliers. A comparison of numerical data between the two independent groups with one independent variable was performed using the two-sample *t*-test. For two independent variables with and without paired observations, a two–way repeated measures or ordinary two-way ANOVA with Holm–Sidak’s multiple comparisons test was used to compare numerical data. Categorical variables were analyzed using Chi-square test followed by the Fisher’s exact test, and survival plots were analyzed using the Log-rank test. Spearman and Pearson correlations were used to assess the linear relationship between ordinal and numerical variables. A *p*-value of ≤0.05 was considered statistically significant.

## 3 Results

### 3.1 Effect of LDC alcohol feeding on survival and blood alcohol levels

The experimental design of alcohol feeding using LDC in aged WT and Tg2576 mice is shown in Figure 1a. No significant changes to mouse average body weights (34.0 ± 1.46 in the alcohol-fed Tg2576 mice vs. 33.9 ± 2.21 in the control-fed Tg2576 mice and 38.2 ± 1.64 in the alcohol-fed WT mice vs. 43.2 ± 2.40 in the control-fed WT mice) were observed between the control-diet- and alcohol-diet-fed groups throughout the study. Average daily food consumption per mouse was monitored between control-diet-fed and alcohol diet-fed mice as per published protocol (Bertola et al., 2013). The average feed consumed per mouse by aged Tg2576 and WT mice was similar, with no significant difference between the control- and alcohol-fed mice (Supplementary Figure 1a). On normalizing the feed consumption by body weight, no significant effect of treatment (control feed and ethanol feed) [*F*_(1,11)_ = 0.25, *p* = 0.62] or sex (males and females) [*F*_(1, 11)_ = 0.1, *p* = 0.76], and no significant interaction between sex and treatment [*F*_(1, 11)_ = 0.18, *p* = 0.68] was observed in the Tg2576 mice (Supplementary Figure 1b). Similarly, normalizing the feed consumption by body weight in WT mice showed no significant effect of sex (males and females) [*F*_(1, 18)_ = 0.026, *p* = 0.87] and no significant interaction between sex and treatment (control feed and ethanol feed) [*F*_(1, 18)_ = 0.028, *p* = 0.87], but a significant treatment effect was observed [*F*_(1, 18)_ = 11.2, *p* = 0.004]. However, post-hoc analysis showed no significant change in the daily food consumption/g body weight between control-diet-fed WT mice and alcohol-diet-fed WT mice (*p* = 0.11) for male and female mice (Supplementary Figure 1c).

Chronic alcohol feeding in the Tg2576 mice significantly reduced survival (*p* = 0.054) compared with the control-diet-fed Tg2576 mice (Figure 1b). The median survival for the alcohol-fed Tg2576 mice was 14 days, and 46% of the mice from this group survived till the end of the study compared with the control-diet-fed Tg2576 mice group in which 83% of the mice survived till the end of the study. Alcohol consumption did not alter the survival of aged WT mice (90 and 93% of mice survived from the control-diet- and alcohol-diet-fed groups at the end of the study, respectively; Figure 1c), suggesting alcohol-induced mortality was specific for aged Tg2576 mice. The blood alcohol concentrations at 5 h after alcohol diet were 11.8 ± 5.1 mg/dL and 9.01 ± 4.82 mg/dL for Tg2576 mice and WT mice, respectively, and are comparable to alcohol levels that result in a mild effect in humans (Jones, 2024).

### 3.2 Effect of alcohol feeding on exploration and anxiety-like behaviors

**Figure 2:**
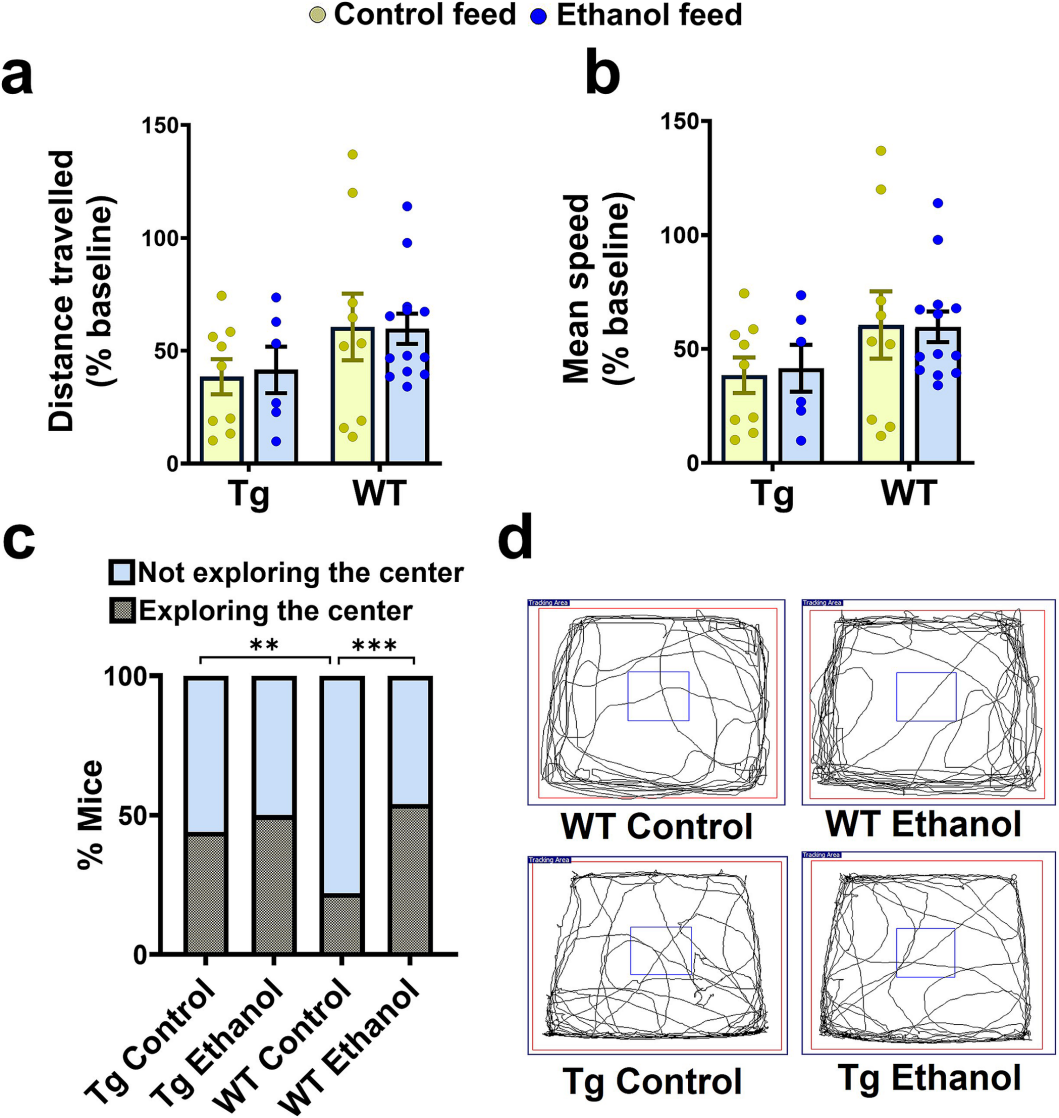
Effect of chronic alcohol-LDC feeding on the exploratory behavior of Tg2576 and wild-type (WT) mice in the open field test. No significant change in the total distance traveled **(a)**, and mean speed **(b)** between the control-diet-fed and chronic alcohol-LDC-diet-fed Tg2576 mice and WT littermates. Alcohol-LDC-diet-fed Tg2576 mice showed no preference to explore the center of the open-field arena compared with the control-diet-fed Tg2576 mice **(c)**. Alcohol-LDC-diet-fed WT mice showed a greater preference to explore the center of the open-field arena than the control-diet-fed WT mice **(c)**. Representative open-field trajectory maps are shown in **(d)**. Data are represented as mean ± SEM of *n* = 6–13 per group and were analyzed using the two-way ANOVA and Holm-Sidak’s post-doc test in **(a,b)**. Chi-square test followed by Fisher’s exact test was used to compare the % of mice in **(c)**. χ^2^_(3)_ = 25,**p < 0.01, ***p < 0.001.

Next, we evaluated the effect of chronic moderate alcohol feeding on the exploration and anxiety of aged Tg2576 mice and their respective WT littermates using the open-field test. Alcohol feeding did not alter the exploratory behavior of either Tg2576 or WT mice (Figures 2a,b,d). Two-way ANOVA showed no effect of mouse genotype (Tg2576 and WT) [*F*_(1, 33)_ = 3.78, *p* = 0.06] or treatment (control feed and ethanol feed) [*F*_(1, 33)_ = 0.01, *p* = 0.92], and no significant interaction between genotype and treatment [*F*_(1, 33)_ = 0.03, *p* = 0.85] for distance traveled and mean speed. No significant difference was observed in the number of mice exploring the center arena between alcohol-fed and control-diet-fed Tg2576 mice (Figure 2c). However, alcohol-fed WT mice showed an increased tendency to explore the center of the open field arena compared to the control-diet-fed WT mice (*p* < 0.001, Figure 2c) suggestive of reduced anxiety in alcohol-fed WT mice compared with control WT mice. Notably, control-diet-fed Tg mice showed an increased tendency to explore the center of the open field arena compared to the control-diet-fed WT mice (*p* < 0.01, Figure 2c), indicating reduced anxiety in the alcohol-fed Tg mice compared to WT mice. Representative trajectory maps showing the mouse movement in the open-field arena are shown in Figure 2d.

### 3.3 Alterations in spatial reference memory by LDC alcohol feeding

**Figure 3:**
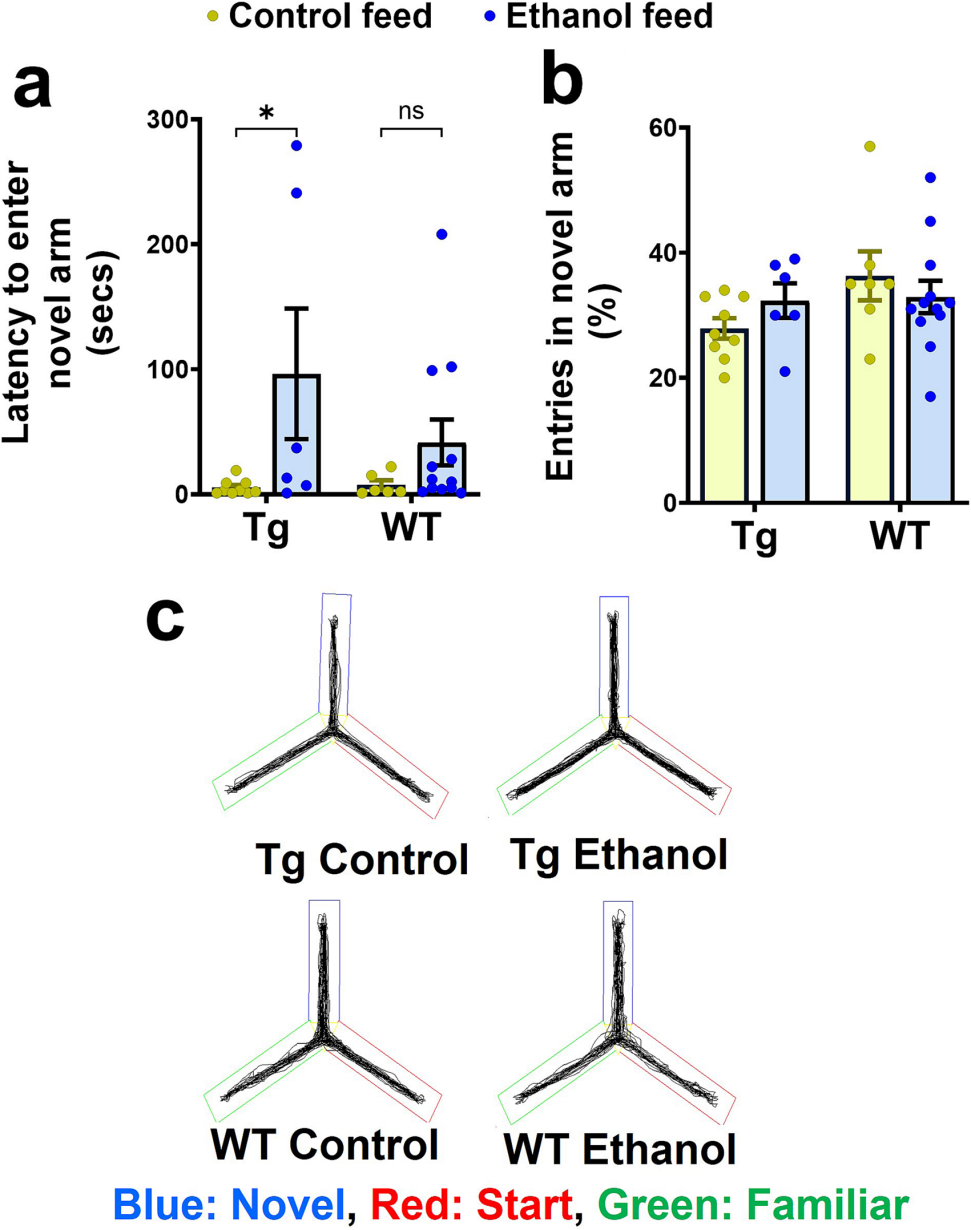
Effect of chronic alcohol-LDC feeding on the spatial reference memory of Tg2576 mice and wild-type (WT) littermates using the Y-maze test. A significant increase in the latency to enter the novel arm was observed for the Tg2576 mice chronically fed with the LDC diet, but no significant change was seen in the WT mice **(a)** compared to their respective control group. No significant change was observed in % entries in the novel arm between the chronic alcohol-LDC-diet-fed and control-diet-fed Tg2576 and WT mice **(b)**. Representative Y-maze trajectory maps are shown in **(c)**. Two mice on the control diet and one mouse on the alcohol-LDC diet from the WT group did not move during the testing phase and were excluded from the analysis. Data are presented as mean ± SEM of *n* = 6–13 per group, and data were analyzed using the two-way ANOVA and Holm-Sidak’s post-doc test in **(a,b)**. *p < 0.05.

To assess the effect of alcohol feeding on spatial reference memory, mice were subjected to the Y-maze test (Ou et al., 2022). Two-way ANOVA revealed no effect of mouse genotype (Tg2576 and WT) [*F*_(1, 29)_ = 1.2, *p* = 0.28] and no significant interaction between mouse genotype and treatment (control feed and ethanol feed) [*F*_(1, 29)_ = 1.4, *p* = 0.25], but a significant effect of treatment [*F*_(1, 29)_ = 6.8, *p* = 0.01] on the latency to the novel arm during the Y-maze test. Post-hoc analysis showed that chronic alcohol feeding significantly increased the latency to enter the novel arm compared with the control diet in the aged Tg2576 mice (*p* < 0.05, Figure 3a). This increase in latency with alcohol feeding was not statistically significant in the aged WT mice (Figure 3a), although a similar trend was observed. Combining Tg2576 and WT mice showed a significant treatment effect such that alcohol-treated mice exhibited increased latency to enter the novel arm compared with the control-diet-fed mice (Supplementary Figure 2). Chronic alcohol or control diet feeding [*F*_(1, 30)_ = 0.03, *p* = 0.85] or mouse genotype [*F*_(1, 30)_ = 2.5, *p* = 0.13] did not significantly affect the number of entries to the novel arm (Figures 3b,c). Further, no significant interaction effect between treatment and mouse genotype was observed for the number of entries to the novel arm [*F*_(1, 30)_ = 1.9, *p* = 0.18]. Representative trajectory maps showing the mouse movement in the Y-maze arena are shown in Figure 3c.

### 3.4 Effect of LDC alcohol feeding on microgliosis and neuroinflammation

**Figure 4:**
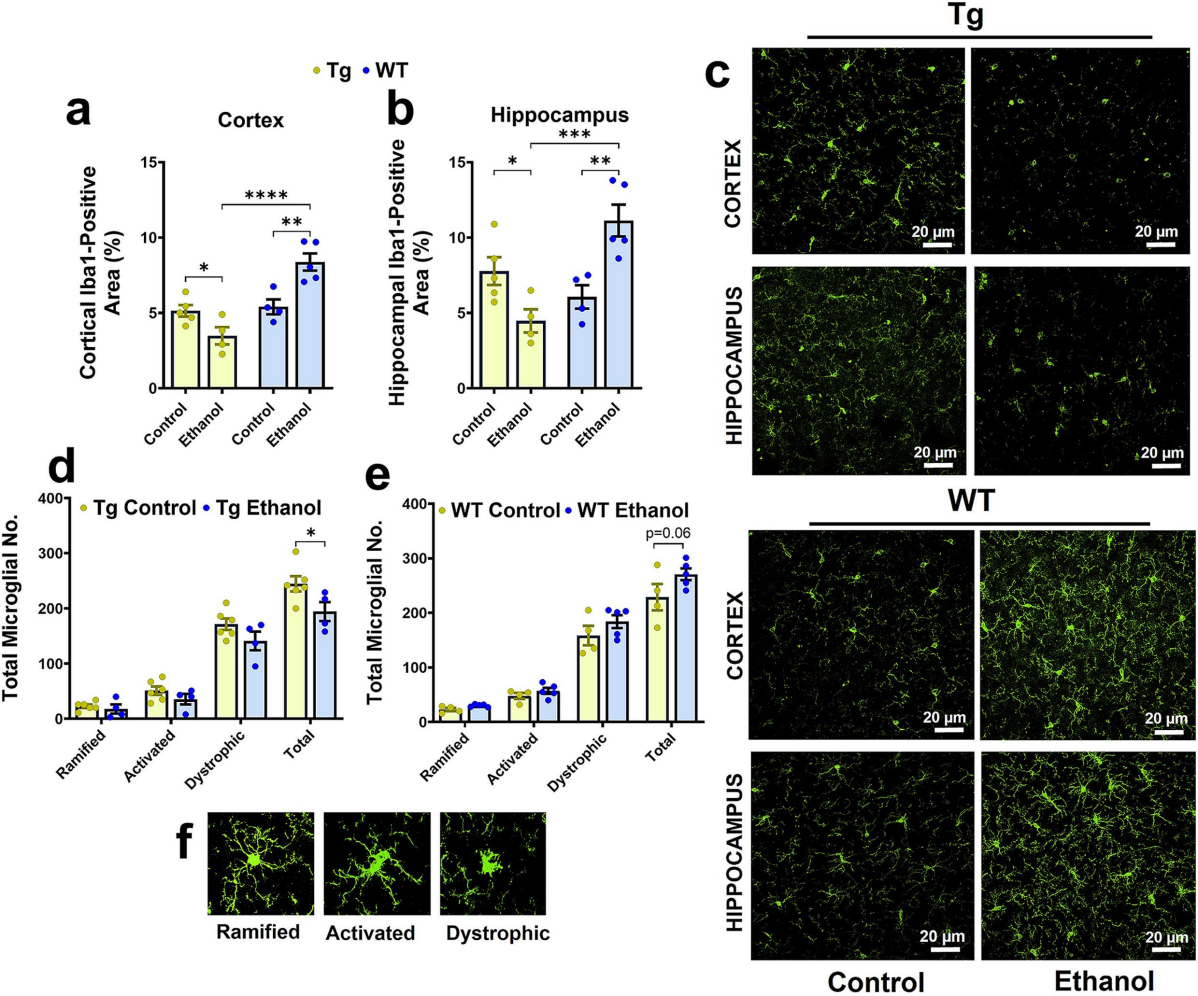
Effect of chronic alcohol-LDC feeding on microgliosis in the brains of male Tg2576 and wild-type (WT) littermates. Cortical **(a)** and hippocampal **(b)** Iba-1-positive-area was significantly reduced and increased in the alcohol-LDC-diet-fed male Tg2576 and WT mice, respectively, compared to their respective control-diet-fed mice. Representative Iba1-positive stains in the cortex and hippocampus of Tg2576 and WT mice with and without alcohol-LDC feeding **(c)**. LDC alcohol-fed Tg2576 male mice showed a significant decrease in the total microglia number **(d)**. A trend toward an increase in the total microglia number was seen between alcohol- and control-diet-fed WT male mice **(e)**. Representative images showing the different microglial subtypes in **(f)**. Data are presented as mean ± SEM of *n* = 4–6 male mice per group and were analyzed using the two-way ANOVA and Holm-Sidak’s post-doc test in **(a,b,d,e)**. *p < 0.05, **p < 0.01,***p < 0.001, and ****p < 0.0001. Scale bar = 20 μm.

Previously, we observed that intragastric alcohol feeding for six weeks to young C57BL6 mice decreased microglial numbers (Iba-1-stained cells, a marker for microglial activation) in the cortex, with a similar trend in the hippocampus (Garcia et al., 2022). Herein, a two-way ANOVA revealed a significant interaction between mouse genotype (Tg2576 and WT) and treatment (control feed and ethanol feed) [*F*_(1, 14)_ = 20.4, *p* = 0.0005] and a significant effect of mouse genotype [*F*_(1, 14)_ = 25.4, *p* = 0.0002] on cortical Iba-1-positive area (Figure 4a). A similar effect was observed in the hippocampus, and a two-way ANOVA revealed a significant interaction between mouse genotype and treatment [*F*_(1, 14)_ = 20.4, *p* = 0.0005] and a significant effect of mouse genotype [*F*_(1, 14)_ = 7.1, *p* = 0.02] on hippocampal Iba-1-positive area (Figure 4b). A post-hoc analysis showed that chronic alcohol-LDC feeding to aged Tg2576 male mice caused a significant reduction in the cortical and hippocampal Iba-1-positive area in the Tg2576 male mice (*p* < 0.05, Figures 4a–c). However, in WT male mice, chronic alcohol-LDC feeding resulted in a significant increase in the cortical and hippocampal Iba-1-positive area (*p* < 0.01, Figures 4a–c). Iba-1-positive area was significantly lower (*p* < 0.001) in the chronic alcohol-LDC-fed Tg2576 mice compared to alcohol-LDC-fed WT mice in the cortex and hippocampus (Figures 4a–c). No significant change in the Iba-1-positive area was observed with chronic alcohol feeding in Tg2576 or WT female mice compared with their respective controls (data not shown).

In addition, we also quantified the total microglia number and their morphological subtypes (ramified, activated, and dystrophic). In the Tg2576 mice, there was a significant effect of microglia morphology subtypes [*F*_(3, 32)_ = 139, *p* < 0.0001] and treatment (control feed and ethanol feed) [*F*_(1, 32)_ = 9.9, *p* = 0.003], but no significant interaction between microglia morphology and treatment [*F*_(3, 32)_ = 1.5, *p* = 0.23], on microglial numbers. Post-hoc analysis showed a significant decrease (*p* < 0.05) in total microglial number (Figure 4d) with chronic alcohol feeding in Tg2576 male mice compared with their control group. A similar trend was observed for the different microglial morphological subtypes (ramified, activated, and dystrophic) in the chronic alcohol-fed Tg2576 male mice, without statistical significance. Similarly, in the WT mice, there was a significant effect of microglia morphology [*F*_(3, 28)_ = 159, *p* < 0.0001] and treatment (control feed and ethanol feed) [*F*_(1, 28)_ = 6.4, *p* = 0.02], but no significant interaction between microglia morphology and treatment [*F*_(3, 28)_ = 0.96, *p* = 0.42], on microglia numbers. Chronic moderate-alcohol feeding showed a trend toward an increase in the number of microglia in the male WT mice (*p* = 0.06, Figure 4e). Representative images of the microglial morphology subtypes are shown in Figure 4f. Additionally, we measured the levels of tumor necrosis factor-alpha (TNF-*α*), which is secreted by microglia, and found that chronic moderate alcohol feeding to aged Tg2576 and aged WT mice did not alter the TNF-α levels in the brains (Supplementary Figure 3).

### 3.5 Changes in Aβ load in brain and plasma following LDC alcohol feeding

**Figure 5:**
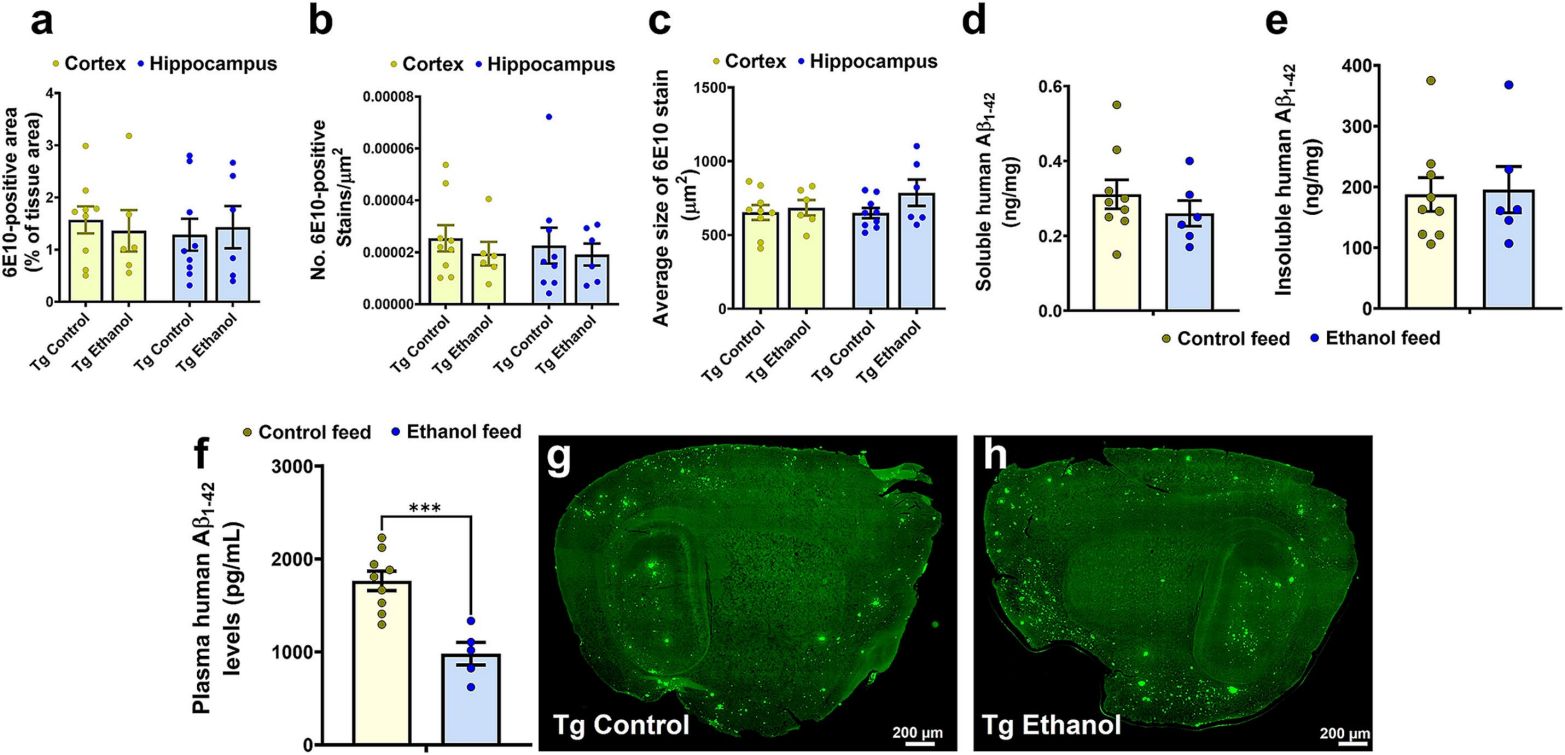
Effect of chronic alcohol-LDC feeding on 6E10-positive human Aβ and human Aβ_1-42_ load in the brains of Tg2576 mice. No significant change in the cortical or hippocampal 6E10-positive area **(a)**, puncta count **(b)**, and average stain size **(c)** between the control-diet and alcohol-LDC-diet-fed Tg2576 mice. No significant change in the soluble **(d)** and insoluble **(e)** human Aβ_1-42_ levels in ng/mg protein between the control-diet- and alcohol-LDC-diet-fed Tg2576 mice brain homogenates. A significant decrease in the plasma human Aβ_1-42_ levels was observed between the control-diet- and alcohol-LDC-diet-fed Tg2576 mice **(f)**. Representative sagittal brain section images of 6E10-positive Aβ stains of control-diet-fed Tg2576 mice (Tg control) **(g)** and alcohol-LDC-diet-fed Tg2576 mice (Tg Ethanol) **(h)**. The data are presented as mean ± SEM of *n* = 5–9 per group and were analyzed using the two-way ANOVA and Holm-Sidak’s post-doc test in **(a–c)** and two-sample *t* test in **(d–f)**. ***p < 0.001. Scale bar = 200 μm.

To assess the effect of chronic moderate alcohol feeding on the Aβ pathology in the aged Tg2576 mice, both 6E10-immunostaining and ELISA were used to detect human Aβ. Alcohol feeding resulted in no significant change in the 6E10-positive area (Figure 5a), 6E10 count (Figure 5b), or average 6E10 stain size (Figure 5c) compared with control-diet-fed Tg2576 mice, both in the cortex and the hippocampus. Two-way ANOVA revealed no effect of brain region (cortex of hippocampus) and treatment (control feed or ethanol feed) and no interaction between brain region and treatment on 6E10-positive area, 6E10 count, or average 6E10 stain size [*F*_(1, 26)_ ranged between 0.009 to 2.3, *p* > 0.05 for each comparison]. Consistent with this, no significant changes were observed in the TBS-soluble [*t*_(13)_ = 0.92, *p* = 0.37, Figure 5d] and insoluble [(*t*_(13)_ = 0.17, *p* = 0.86, Figure 5e] human Aβ_1-42_ levels between the alcohol-fed and control-diet-fed Tg2576 mice. There was a significant decrease [*t*_(12)_ = 4.6, *p* < 0.001] in the plasma human Aβ_1-42_ levels in the chronic alcohol-fed Tg2576 mice compared to that of the control group (Figure 5f). Representative 6E10-stained brain sections from control-diet-fed Tg2576 mice and alcohol-fed Tg2576 mice are shown in Figures 5g,h.

**Figure 6:**
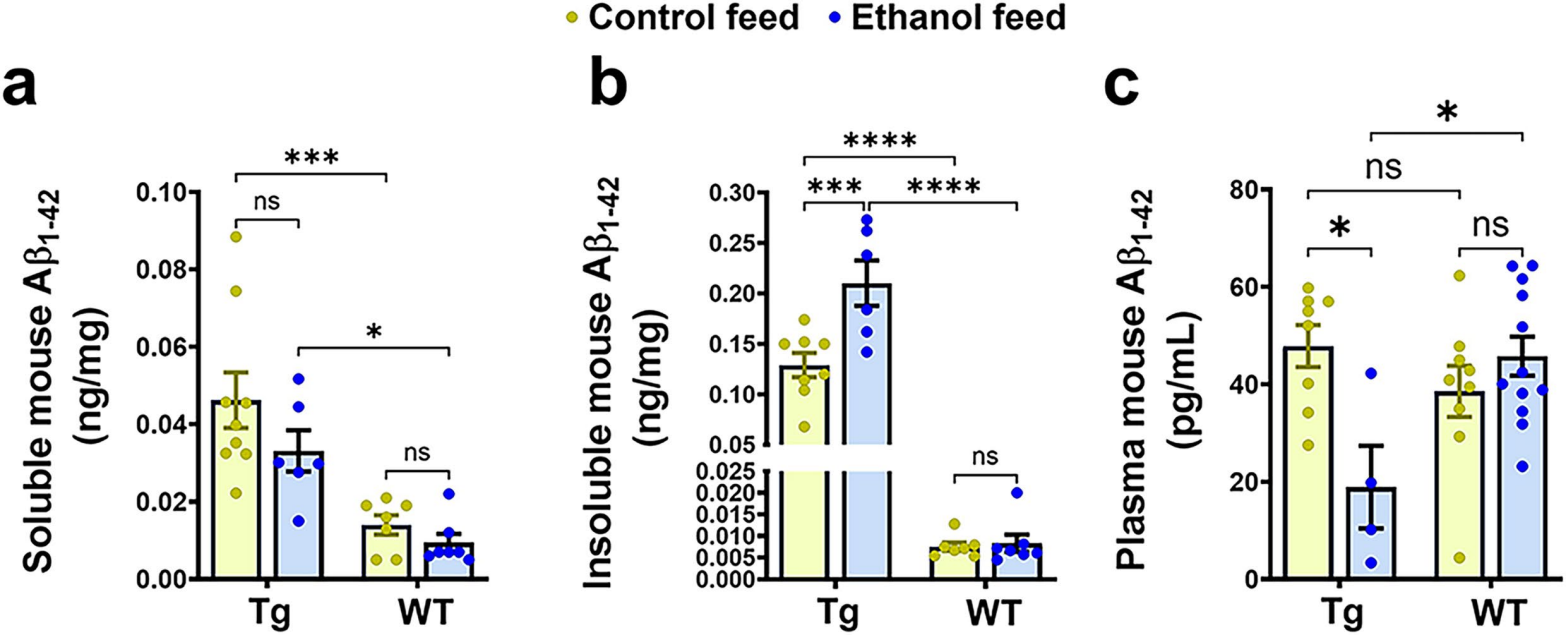
Effect of alcohol-LDC feeding on endogenous mouse Aβ load in the brains of Tg2576 mice and wild-type (WT) littermates. The TBS-soluble mouse Aβ_1-42_ levels in ng/mg protein in the brain samples were significantly altered between the mouse genotypes (Tg2576 and WT) but no significant change was observed between control or alcohol-LDC diet fed mice **(a)**. Endogenous mouse insoluble Aβ_1-42_ levels in ng/mg protein in the brain samples were significantly altered between the mouse genotypes and treatments. A significant increase was seen in the endogenous mouse insoluble Aβ_1-42_ levels in the brain samples of Tg2576 mice fed with alcohol-LDC diet compared with control-diet-fed Tg2576 mice **(b)**. A significant decrease was observed in the plasma endogenous mouse Aβ_1-42_ levels in Tg2576 mice fed with alcohol-LDC diet compared with control-diet-fed Tg2576 mice **(c)**. No changes were seen in the endogenous mouse soluble Aβ_1-42_ **(a)**, insoluble Aβ_1-42_ **(b)**, and plasma Aβ_1-42_ **(c)** levels of the WT mice fed with alcohol-LDC diet compared to control-diet-fed WT mice. Data are presented as mean ± SEM of *n* = 5–13 per group and were analyzed using the two-way ANOVA and Holm-Sidak’s post-doc test. *p < 0.05, ***p < 0.001, and ****p < 0.0001.

We next investigated the effect of LDC alcohol feeding on endogenous mouse brain Aβ_1-42_ levels in both aged Tg2576 and WT mice (Figure 6). Endogenous TBS-soluble mouse Aβ_1-42_ levels were significantly altered by mouse genotype (Tg2576 and WT) [*F*_(1, 25)_ = 27.6, *p* < 0.0001] but were not affected by treatment (control feed and ethanol feed) [*F*_(1, 25)_ = 2.8, *p* = 0.11] (Figure 6a). Further, no interaction effect was observed between mouse genotype and treatment on endogenous TBS-soluble mouse Aβ_1-42_ levels [*F*_(1, 25)_ = 0.64, *p* = 0.43]. Post-hoc analysis showed that TBS-soluble mouse Aβ_1-42_ levels were significantly higher in the Tg2576 mice compared with WT mice in control (*p* < 0.001) and ethanol (*p* < 0.05) fed groups. Endogenous mouse insoluble Aβ_1-42_ levels were significantly affected by mouse genotype [*F*_(1, 24)_ = 186, *p* < 0.0001], treatment [*F*_(1, 24)_ = 11.9, *p* = 0.002], and a significant interaction between mouse genotype and treatment was observed [*F*_(1, 24)_ = 11.5, *p* = 0.002] (Figure 6b). Post-hoc analysis showed that chronic alcohol feeding to Tg2576 mice significantly increased (*p* < 0.001) endogenous mouse insoluble Aβ_1-42_ levels, compared with the control-diet-fed Tg2576 mice (Figure 6b). Endogenous mouse insoluble Aβ_1-42_ levels were significantly higher (*p* < 0.0001) in the Tg2576 mice compared to WT mice with and without alcohol treatment (Figure 6b). For endogenous mouse Aβ_1-42_ plasma levels, there was a significant treatment effect [*F*_(1, 29)_ = 4, *p* = 0.05] and interaction between mouse genotype and treatment [*F*_(1, 29)_ = 11.1, *p* = 0.002] (Figure 6c). Post-hoc analysis showed that endogenous mouse Aβ_1-42_ levels were significantly lower in alcohol-fed Tg2576 mice compared with control-diet-fed Tg2576 mice (*p* < 0.05) and alcohol-fed WT mice (*p* < 0.05) (Figure 6c), similar to plasma human Aβ_1-42_ levels (Figure 5f). It should be noted that since human APP is overexpressed under the prion protein (PrP) promoter in Tg2576 mice, human Aβ levels were several magnitudes greater than mouse Aβ levels in the brain and plasma (Figures 5, 6).

### 3.6 Effect of LDC alcohol feeding on proteins involved in Aβ production, synaptic health, and BBB function

**Figure 7:**
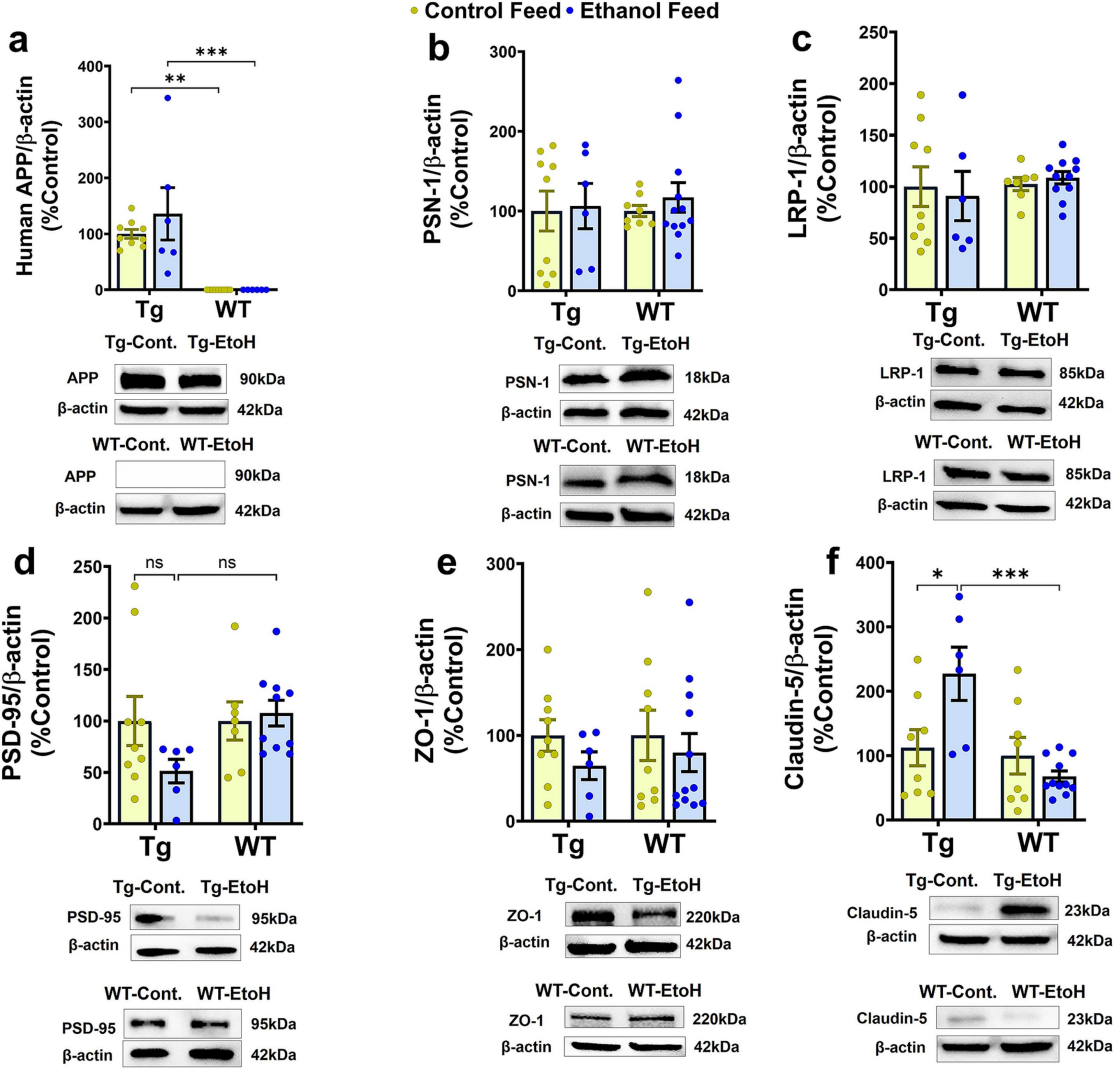
Western blot data for proteins involved in Aβ synthesis and transport, synaptic health, and BBB function in the brains of Tg2576 and their respective wild-type (WT) littermates, with and without alcohol feeding. No significant change was observed in brain homogenate-derived protein levels of APP **(a)**, PSN-1 **(b)**, LRP-1 **(c)**, PSD-95 **(d)**, and ZO-1 **(e)** between the control-diet- and alcohol-LDC-diet-fed Tg2576 and WT mice. Claudin-5 levels were significantly upregulated in chronic alcohol-fed Tg2576 mice brains compared to control-diet-fed Tg2576 mice and alcohol-fed WT mice. No change was observed in the expression levels of claudin-5 between control-diet- and alcohol-LDC-diet-fed WT mice **(f)**. Full-blot images are shown in Supplementary Figure 6. Data are presented as mean ± SEM of *n* = 6–13 per group and were analyzed using the two-way ANOVA and Holm-Sidak’s post-doc test. *p < 0.05, **p < 0.01, and ***p < 0.001.

We next investigated the effect of chronic moderate alcohol feeding on the levels of proteins important in Aβ homeostasis and synaptic health. As expected, WT mice did not express human APP in the brain and human APP expression in the brain differed only by genotype [*F*_(1, 26)_ = 36.9, *p* < 0.0001] (Figure 7a). No treatment (control feed and alcohol feed) effect or interaction between mouse genotype and treatment was observed (Figure 7a), and human APP expression in the brain did not change with alcohol-LDC feeding in Tg2576 mice. This suggests that the expression of human APP under the PrP promoter was not affected by alcohol (Figure 7a). We unfortunately could not find an antibody to specifically detect mouse APP and not human APP; thus, levels of mouse APP were not measured in the brains of aged Tg2576 mice. The expression levels of other proteins involved in Aβ production (presenilin 1 (PSN1) and Aβ clearance (LRP-1)), were also not affected by alcohol feeding to Tg2576 mice or WT mice or mouse genotype (Figures 7b,c). Synaptic health (Post synaptic density-95, PSD-95) and BBB tight-junction protein (zonula occludens, ZO-1) were unchanged by alcohol-LDC feeding to Tg2576 and WT mice and mouse genotype (Figures 7d,e). We observed a significant effect of mouse genotype [*F*_(1, 29)_ = 11.3, *p* = 0.002] and interaction between mouse genotype and treatment [*F*_(1, 29)_ = 8.3, *p* = 0.007] on claudin-5 expression (another BBB tight-junction protein). Post-hoc analysis showed an upregulation of claudin-5 in the chronic alcohol-fed Tg2576 mice brains compared to control-diet-fed Tg2576 mice (*p* < 0.05) and alcohol-fed WT mice (*p* < 0.001) (Figure 7f).

### 3.7 Assessment of liver injury caused by LDC alcohol feeding

**Figure 8:**
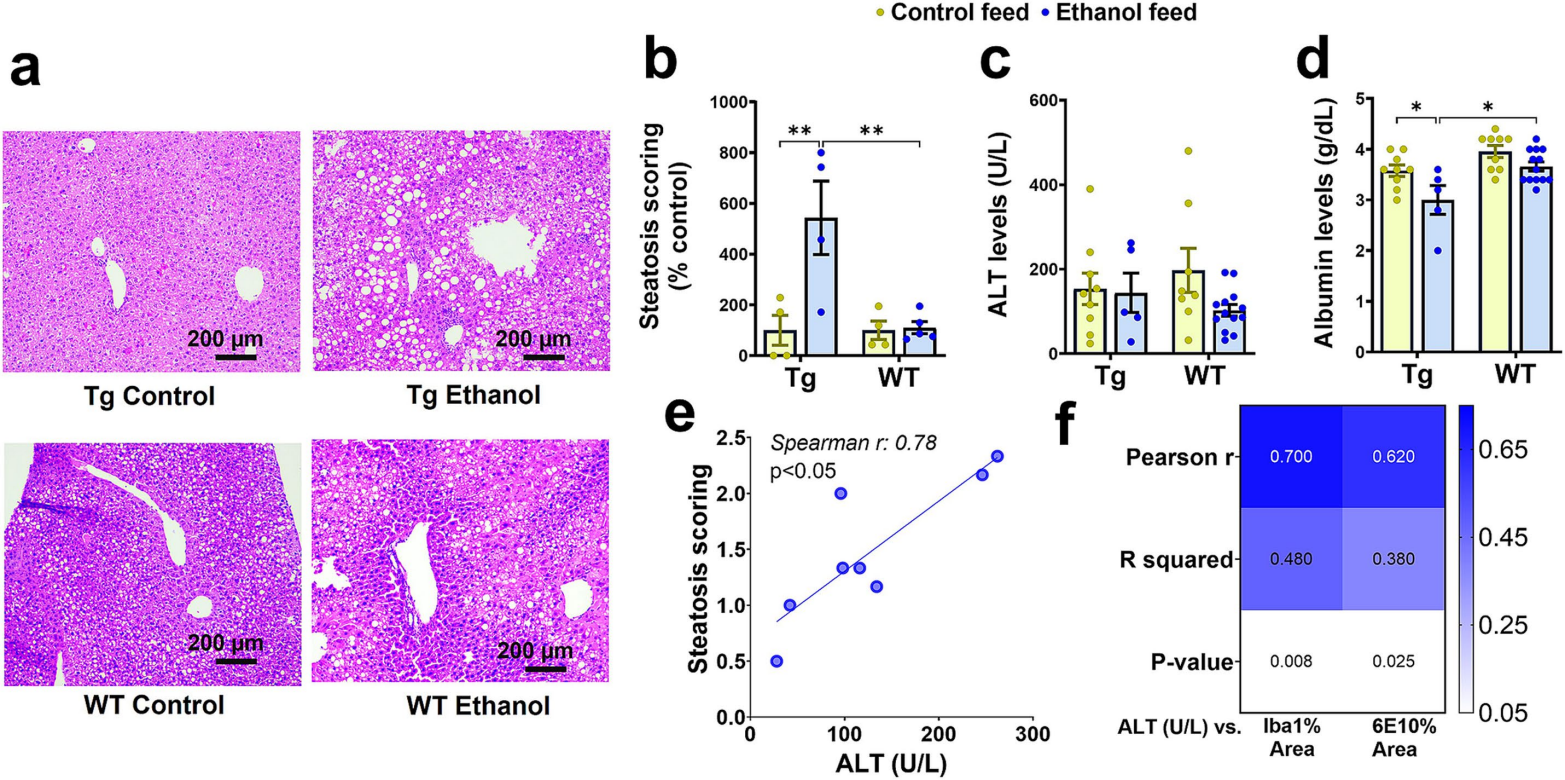
Effect of alcohol-LDC feeding on liver histology and serum biomarkers of hepatic function in Tg2576 and wild-type (WT) mice. Representative photomicrographs of liver histopathology of control- and alcohol-LDC-diet-fed Tg2576 and WT mice stained with hematoxylin–eosin **(a)**. Scale bar = 200 μm. Alcohol-LDC feeding in Tg2576 mice significantly increased the steatosis score compared with control-diet-fed Tg2576 mice and alcohol-fed WT mice **(b)**. No significant change in steatosis score in alcohol-LDC-diet-fed WT mice compared to control-diet-fed WT mice **(b)**. No significant change was observed in the serum alanine aminotransferase (ALT) levels between the control and alcohol-LDC-diet-fed Tg2576 mice and WT mice **(c)**. A significant reduction in the serum albumin levels was observed in alcohol-LDC-diet-fed Tg2576 mice compared to control diet-fed Tg2576 mice and alcohol-fed WT mice **(d)**. No significant change in serum albumin levels in alcohol-LDC-diet-fed WT mice compared with control-diet-fed WT mice **(d)**. Scatter plot showing the correlation between serum ALT and steatosis scoring in alcohol-LDC-diet-fed Tg2576 and WT mice combined **(e)**, and heat-map showing the correlations between serum ALT and 6E10 area and Iba1 area in Tg2576 mice **(f)**. Data are presented as mean ± SEM of *n* = 4–13 per group in b-d, and data were analyzed using the two-way ANOVA with the Holm-Sidak’s post-hoc test in **(b-d)** and the Spearman and Pearson correlation in **(e,f)**, respectively. *p < 0.05 and **p < 0.01.

As previously mentioned, chronic LDC-alcohol feeding is generally considered a model of moderate alcohol drinking based on liver injury (Bertola et al., 2013). Measurement of various liver injury markers shows some extent of liver injury with LDC-alcohol feeding in aged Tg2576 mice. Liver histology demonstrated a significant effect of mouse genotype [*F*_(1, 13)_ = 7.99, *p* = 0.01], treatment [*F*_(1, 13)_ = 8.8, *p* = 0.01], and a significant interaction between mouse genotype and treatment [*F*_(1, 13)_ = 7.9, *p* = 0.01], on liver steatosis. Post-hoc analysis showed increased steatosis (fatty liver) with alcohol feeding in aged Tg2576 mice, which, when scored by a blinded reader, was found to be significantly increased over the control diet-fed Tg2576 mice (*p* < 0.01) and alcohol-fed WT mice (*p* < 0.01) (Figures 8a,b). LDC-alcohol feeding to aged WT mice did not cause a significant increase in steatosis scoring (Figures 8a,b). No significant differences were observed in the serum ALT levels of alcohol-LDC diet-fed and control-LDC diet-fed Tg2576 and WT mice (Figure 8c). However, another marker of liver injury, serum albumin, was significantly altered by mouse genotype [*F*_(1, 32)_ = 14.5, *p* = 0.0006] and treatment [*F*_(1, 32)_ = 10.2, *p* = 0.003], with no interaction between mouse genotype and treatment. Post-hoc analysis showed decreased serum albumin in alcohol-LDC diet-fed Tg2576 mice compared to control diet-fed Tg2576 mice (*p* < 0.05) and alcohol-fed WT mice (*p* < 0.05) (Figure 8d). No significant change in the serum albumin levels was seen with alcohol feeding to WT mice (Figure 8d), suggesting WT mice suffered less liver injury with alcohol feeding compared to Tg2576 mice. Steatosis score was significantly positively correlated with serum ALT values in alcohol-fed Tg2576 and WT mice combined (*p* < 0.05, Spearman r = 0.78, Figure 8e). Finally, we examined if liver injury was a significant correlate of AD pathology. Serum ALT levels were significantly correlated with Iba1-positive- and 6E10-positive-area in the Tg2576 mice (Figure 8f). No significant changes were seen in other liver injury biomarkers measured in the serum samples of the alcohol-LDC diet-fed Tg2576 and WT mice compared to their respective control-LDC diet-fed groups, but a trend toward an increase in liver/body weight ratio was seen in the alcohol-fed Tg2576 mice (Supplementary Figure 4).

### 3.8 Effect of LDC alcohol feeding on hepatic Aβ and Aβ-relevant proteins

**Figure 9:**
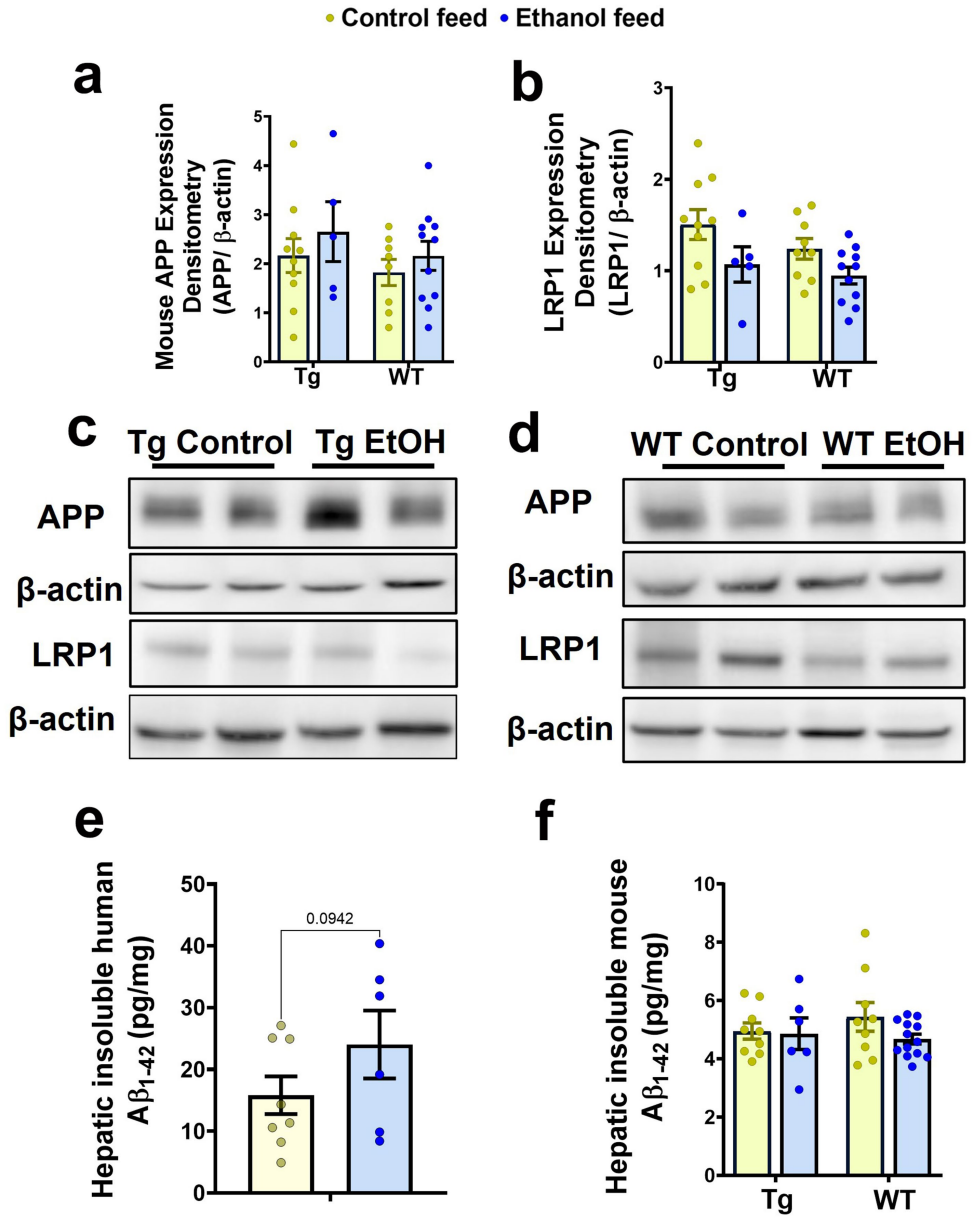
Effect of alcohol-LDC feeding on hepatic Aβ levels and the expression levels of proteins relevant to Aβ synthesis and transport in the livers of Tg2576 and wild-type (WT) mice. Protein expression levels of mouse APP in the liver samples by Western blot showed no significant change in Tg2576 and WT mice with and without alcohol feeding **(a,c,d)**. A trend toward a decrease in the expression of hepatic LRP-1 was observed with alcohol feeding in the Tg2576 mice and WT mice **(b,c,d)**. β-actin was used as the loading control. Full-blot images are shown in Supplementary Figure 7. Chronic alcohol-LDC feeding did not significantly change human insoluble hepatic Aβ_1-42_ expressed in pg/mg protein in Tg2576 mice but a trend toward an increase was observed **(e)**. Endogenous mouse insoluble hepatic Aβ_1-42_ levels expressed in pg/mg protein were unchanged in Tg2576 and WT mice with and without alcohol feeding **(f)**. Data are presented as mean ± SEM of *n* = 5–13 per group, and data were analyzed using the two-way ANOVA with the Holm-Sidak’s post-hoc test.

Previously we demonstrated that chronic alcohol feeding using intragastric feeding or the NIAAA bolus feeding models, methods that cause greater liver injury than chronic LDC alcohol feeding, increased hepatic APP and decreased hepatic LRP-1 in 8-week-old (young) WT mice and decreased hepatic LRP-1 in 7-month-old APP/PS1 mice (Garcia et al., 2022; Chandrashekar et al., 2024). Since hepatic APP and LRP-1 can affect peripheral and brain Aβ (Garcia et al., 2022, Chandrashekar et al., 2024), we similarly examined if hepatic levels of these key proteins were altered in aged Tg2576 and WT mice following chronic LDC-alcohol feeding. Immunoblotting demonstrated no change in mouse APP with mouse genotype or treatment (Figures 9a,c,d). With respect to hepatic LRP-1, a significant treatment (control feed and alcohol feed) effect was observed [*F*_(1, 31)_ = 6.6, *p* = 0.01], and a small trend toward an alcohol-mediated decrease in LRP-1 in the livers of Tg2576 and WT mice was seen (Figures 9b,c,d). The antibody used to detect APP in the liver cross-reacts with both human and mouse APP. However, since human APP is largely expressed in the brains of the Tg2576 mice, APP detected in the liver is likely endogenous mouse APP. qPCR measurements also confirmed that hepatic APP and LRP-1 changes were not significantly altered by LDC alcohol feeding (Supplementary Figure 5).

Since mouse hepatic APP can be a source of mouse Aβ_1-42_ in the plasma, and hepatic LRP-1 may take up both mouse Aβ_1-42_ and human Aβ_1-42_ from plasma, the levels of both human and mouse Aβ_1-42_ in the livers of Tg2576 mice, and mouse Aβ_1-42_ in the livers of WT mice (since WT mice have no human APP), were measured. No significant change was observed in the insoluble human Aβ_1-42_ levels in the liver with chronic alcohol feeding of Tg2576 mice, though a trend toward an increase was observed (Figure 9e). Similarly, endogenous hepatic mouse insoluble Aβ_1-42_ levels did not change in both Tg2576 mice and WT mice with chronic alcohol feeding compared to their respective control diet-fed groups (Figure 9f).

## 4 Discussion

### 4.1 Aged Tg2576 mice are susceptible to chronic LDC alcohol feeding

In this study, we examined the effect of chronic alcohol feeding using the LDC liquid diet (5% v/v for 6 weeks) on AD pathology in 13-month-old Tg2576 AD and WT mice. LDC alcohol feeding allows for nutritional balance and is generally associated with liver pathology that corresponds with low-moderate alcohol drinking or the early stages of liver disease (Brandon-Warner et al., 2012). LDC alcohol feeding generally reduces liquid diet consumption in alcohol-fed mice due to their aversion to alcohol. Therefore, liquid diet intake of the control mice is adjusted to match that of the LDC alcohol-fed mice (Brandon-Warner et al., 2012). Liver pathology measurements (e.g., steatosis, ALT, and albumin values) suggest that LDC alcohol feeding to aged WT mice caused negligible liver injury (Figure 8). However, in aged Tg2576 AD mice, this model of alcohol feeding was associated with high mortality and liver injury (Figures 1, 8). Only 46% of LDC alcohol-fed aged Tg2576 mice survived compared to 83% for aged control Tg2576 mice (Figure 1). Since WT mice of similar age did not suffer high mortality with LDC alcohol feeding (90 and 93% of the mice survived in the control-diet- and alcohol-fed groups, respectively, Figure 1), the alcohol-induced mortality appears to be specific for aged Tg2576 AD mice. Why the aged Tg2576 AD mice with amyloid plaques are more sensitive to alcohol-induced mortality remains unknown. In young and lightweight mice, chronic alcohol feeding can cause mortality due to hypothermia caused by alcohol (Bertola et al., 2013; Guo et al., 2018). Daily temperature monitoring was not performed in the current study, and it is unclear if AD pathology or AD-induced behavior changes promote hypothermia, which contributed to increased mortality in the aged Tg2576 mice. Since we experienced high mortality with alcohol feeding in aged Tg2576 mice, we need to consider a survivor effect in our study. The possibility that the alcohol-fed Tg2576 mice survivors have some characteristics associated with survival that may bias the data must be acknowledged.

### 4.2 Effect of chronic alcohol feeding on cognition and behavior in aged Tg2576 mice

In our study, chronic alcohol feeding to Tg2576 mice did not cause any significant changes in the locomotion or anxiety-related phenotypes as tested by the open field test (Figure 2). However, we observed that alcohol-fed WT mice showed an increased tendency to explore the center of the open field indicating reduced anxiety with moderate alcohol feeding, which has been shown in ethanol-fed rats (Blokland et al., 1992). This increase in exploration of the center arena is not confounded by increased movement as the indices of locomotion remained unchanged in alcohol-LDC-fed WT mice compared to controls. In contrast to our findings, a recent study showed that chronic moderate alcohol feeding (alcohol in drinking water using the two-bottle choice paradigm) to 5.5-month-old APP/PS1 mice increased locomotor activity and central zone exploration in the open field test with no significant effect on their respective alcohol-fed WT littermates. This indicates that alcohol feeding may have varying effects on behavioral paradigms due to alcohol feeding techniques, mouse models, or the age of mice (Day et al., 2023).

Chronic alcohol-LDC feeding appeared to affect memory in aged Tg2576 mice but not in aged WT mice. During the Y-maze test used to assess spatial reference memory, chronic alcohol-LDC feeding to the Tg2576 mice increased the latency to enter the novel arm indicating that alcohol negatively impacts learning and memory in the Tg2576 mice (Figure 3). Our findings of cognitive impairment in aged AD mice are consistent with other alcohol-feeding studies in young AD mice. Chronic alcohol exposure to young 3xTg-AD mice through vapors, oral gavage, or by the two-bottle choice paradigm model caused cognitive impairment as assessed by the Morris water maze (Hoffman et al., 2019; Barnett et al., 2022; Sanna et al., 2023). Apart from anxiety and spatial memory deficits, AD mice show impairment in fear conditioning, which is exacerbated with alcohol intake (Hoffman et al., 2019). Fear conditioning was not studied herein, and future work is needed to determine the effect of chronic moderate LDC alcohol feeding on contextual and cued fear learning in AD mice. Taken together, our data and other work support the notion that chronic alcohol intake accelerates cognitive impairment associated with AD in both young and aged AD mice.

### 4.3 Microglial alterations caused by chronic LDC alcohol feeding

Microglia are the neuroimmune cells of the central nervous system that are activated in response to pathological stimuli and help in the phagocytic removal of pathogens and other cell debris, including Aβ plaques (Leng and Edison, 2021). Microglia may play both a protective and pathogenic role in AD pathology. On one hand, microglia associated with Aβ plaques can restrict their further expansion and protect from neuronal injury and/or clear Aβ deposits in the AD brain (Spangenberg et al., 2019). On the other hand, the repeated activation of microglia can release excessive pro-inflammatory mediators, which can aggravate AD pathology (Spangenberg et al., 2019; Cai et al., 2022). It has been speculated that the toxic effects produced by chronic alcohol consumption are too mild to result in noticeable microglial activation in the brain (Streit et al., 1988); however, some studies report that alcohol drinking could trigger microglial activation through toll-like receptors (TLRs) and cytokine signaling (Crews et al., 2017) and cause functional deficits (Marshall et al., 2020; Lowe et al., 2020). Further, alcohol treatment of microglia in cell culture was shown to reduce Aβ phagocytosis (Kalinin et al., 2018). We studied microgliosis in the cerebral cortex and the hippocampal regions of the brain, which are known to be affected by chronic alcohol consumption (Fructuoso et al., 2020; Cruz et al., 2017). We observed that chronic alcohol feeding to male Tg2576 mice caused a significant reduction in microgliosis (Iba1-positive area), whereas alcohol feeding to male WT littermates caused a significant increase in Iba1-positive area (Figure 4). Previously, we observed that alcohol feeding to young male WT mice by intragastric feeding, that causes greater liver injury than LDC diet, decreased microgliosis. A reduction in microglial numbers and increased microglial dystrophy is reported with binge alcohol feeding in adolescent and adult (postnatal day 35–70) male rats (Marshall et al., 2020), supporting our findings of reduced microgliosis in alcohol-fed aged Tg2576 male mice. The increased microgliosis in aged alcohol-fed WT mice, though in contrast to some previous work (Grifasi et al., 2019), may be attributed to age-associated microglial priming (Perry and Holmes, 2014). It is conceivable that aging exacerbates the effect of alcohol on microglia in WT mice, and this response may be dysfunctional in aged Tg2576 mice. To further evaluate if this increase in microglial immunoreactivity in the aged alcohol-fed WT male mice was associated with increased pro-inflammatory response, we measured TNF-*α* in brain homogenates. However, in our current study, chronic moderate alcohol feeding did not increase TNF-α levels in brain homogenates of Tg2576 mice and their respective WT mice compared to controls at the time of sacrifice (Supplementary Figure 3). The effect of alcohol feeding on other pro-inflammatory cytokines was not studied and cannot be ruled out. In addition, although the number of female Tg2576 mice was low and did not allow for sex-specific comparisons, the observation that alcohol-LDC diet-fed WT female mice did not show any significant change in microgliosis is supported by a report showing that female C57BL/6 J mice subjected to chronic binge alcohol feeding had several folds lower differentially expressed proteins in microglia compared to male mice, showing reduced microglial activity in the alcohol-fed female mice (Rath et al., 2020).

### 4.4 Effect of chronic LDC alcohol feeding on mouse and human Aβ load in the brain

Aβ_1–42_ peptide is considered the toxic form of Aβ, and its accumulation is the precursor to plaque formation (Billings et al., 2005), one of the main neuropathological features of AD. Chronic alcohol consumption can directly affect Aβ production by modulating proteins (APP and PSN-1) involved in its synthesis and thereby aggravating AD pathology (Gong et al., 2021; Kim et al., 2011). In the current study, chronic moderate alcohol feeding did not show any significant effect on human Aβ load in the Tg2576 mice, as confirmed with no change in the 6E10-positive staining (Figure 5). Similarly, no significant change was observed in the soluble and insoluble human Aβ_1-42_ levels with chronic LDC alcohol feeding to Tg2576 mice (Figure 5). Further, no change was observed in the human APP or PSN-1 protein expression in brain homogenates of alcohol-LDC-fed Tg2576 mice compared to control mice (Figure 7). These data suggest that overexpressed human APP under the control of the PrP promoter (Sasaguri et al., 2017; Sturchler-Pierrat et al., 1997) is not induced by alcohol feeding and that there is no increased PSN-1 to increase APP cleavage in the Tg2576 mice in our study.

In contrast to the above findings, we observed that chronic alcohol-LDC feeding to the aged Tg2576 mice increased endogenous mouse insoluble Aβ_1-42_ levels in the brain with a concomitant decrease in plasma mouse Aβ_1-42_ (Figure 6). There was no significant change in the endogenous mouse Aβ_1-42_ levels in the WT mice fed with chronic alcohol-LDC diet. The extent to which mouse insoluble Aβ_1-42_ levels in the brain contribute to AD pathology in the Tg2576 mice may be small, given that the levels of insoluble mouse Aβ_1-42_ were ~ 1,000-fold lower than human Aβ_1-42_ (Figures 5, 6). The endogenous APP promoter contains a heat shock element that can be upregulated by alcohol in cells (Dewji et al., 1995; Dewji and Do, 1996) and may explain the upregulation of mouse Aβ_1-42_ in alcohol-LDC-fed Tg2576 mice in our studies. Unfortunately, we could not find a specific antibody against mouse APP to determine if increased expression of mouse APP was responsible for the increased mouse insoluble Aβ_1-42_ levels in the brain. The increase in mouse brain Aβ_1-42_ may be driven from the periphery as we observed a decrease in plasma mouse Aβ_1-42_ levels in alcohol-LDC fed Tg2576 mice. In contrast, some other studies involving chronic alcohol feeding to transgenic AD mice have observed increased human Aβ load (Hoffman et al., 2019; Huang et al., 2018), suggesting alcohol delivery, AD mice, and age may be factors in modulating human Aβ load.

### 4.5 Effect of alcohol on BBB tight junction proteins, brain LRP-1, and synaptic proteins

The BBB plays a key role in protecting the brain from infiltrating pathogens and neurotoxic chemicals. The structural integrity of brain microvascular endothelial cells (BMECs) at the BBB is maintained by the tight junction proteins, including claudin-5, occludin, and ZO-1 (Citi and Cordenonsi, 1998; Huber et al., 2001). BBB dysfunction can promote cognitive decline through different mechanisms, including alteration in the function and expression of BBB transporters/receptors and infiltration of peripheral immune cells and neurotoxins, which can aggravate AD pathology (Chen et al., 2023). Studies using cultured brain endothelial cells suggest that alcohol exposure decreases the expression of BBB tight junction proteins (Haorah et al., 2005). In contrast to these findings, we surprisingly observed a significant increase in the expression of claudin-5 in the brain homogenates of aged Tg2576 mice treated with chronic alcohol-LDC diet (Figure 7). Since no increase in claudin-5 expression was observed in aged WT mice fed with alcohol-LDC diet, the alcohol-induced increase in claudin-5 appears to be Tg2576 specific. Whether increased claudin-5 expression translates into altered, possibly improved, changes in BBB integrity merits further investigation. No changes in ZO-1 expression were observed in Tg2576 and WT mice with alcohol-LDC feeding. Additionally, proteins relevant to Aβ uptake and synaptic health did not show significant changes in the brain with alcohol-LDC feeding in either aged Tg2576 or WT mice (Figure 7). Neuronal LRP-1 plays an important role in Aβ uptake, and LRP-1 expressed at the BBB plays an important role in exporting Aβ out of the brain and into the periphery (Wang et al., 2021; Petralla et al., 2024), mechanisms that regulate brain Aβ levels. Alcohol-LDC feeding did not alter LRP-1 expression in the brain of aged Tg2576 or WT mice, similar to our previous observation in young C57BL/6 mice and APP/PS1 mice fed alcohol intragastrically (Garcia et al., 2022; Chandrashekar et al., 2024). We used PSD-95 as a marker of synaptic health (Hunt et al., 1996), which is known to stabilize glutamate receptors in the post-synaptic membranes (Zhang et al., 2014), and found a trend toward a decrease in the chronic alcohol-LDC-fed Tg2576 mice and not in alcohol-LDC-fed WT mice (Figure 7). Other studies have observed significant declines in PSD-95 with alcohol feeding (Varodayan et al., 2018), which can be associated with the progression of memory deficits in AD mice (Oakley et al., 2006). The modest but significant deficits in the Y-maze (Figure 3) may be explained by the modest reduction in synaptic health in the alcohol-LDC fed Tg2576 mice.

### 4.6 The role of the liver-brain axis in AD pathology

The liver is responsible for many functions necessary for brain homeostasis, including metabolic regulation and removal of toxic molecules such as Aβ from the periphery. Consequently, liver injury and disruption of the liver-brain axis by chronic alcohol intake may play an important role in AD progression. Liver injury observed with LDC liquid diet used to deliver nutritionally controlled alcohol to aged Tg2576 mice resulted in liver pathology associated with mild liver injury: significant liver steatosis and a significant reduction (~13%) in the albumin (Figure 8). Tg2576 mice were more susceptible to alcohol-induced liver injury compared to WT mice (Figure 8). However, no changes in serum ALT levels were seen with alcohol feeding, which is consistent with the absence of ALT and AST increase with chronic LDC alcohol feeding to WT mice (Bertola et al., 2013; Guo et al., 2018). Additionally, AST levels were not measured because elevated AST levels have been observed in non-hepatic disorders (Han et al., 2023). Despite no changes in serum ALT levels with alcohol-LDC feeding, the extent of liver steatosis was significantly correlated with serum ALT levels in the alcohol-LDC fed Tg2576 and WT mice (Figure 8), suggesting that ALT is still a relevant liver injury marker within the alcohol-LDC fed mice. The serum levels of ALT of Tg2576 mice were positively correlated with two AD hallmarks: Aβ load and neuroinflammation (Iba-1) (Figure 8), indicating significant liver-brain crosstalk with chronic LDC alcohol feeding.

Previously, we observed that chronic intragastric alcohol feeding to young C57BL/6 mice resulted in a ~ 50% decrease of LRP-1, the major hepatic receptor responsible for the removal of Aβ from the periphery, and simultaneously caused a 100% increase in APP expression, a potential source of Aβ in the periphery (Garcia et al., 2022). Similarly, we observed a reduction in hepatic LRP-1 and a concomitant increase in brain Aβ with intragastric alcohol feeding to APP/PS1 mice (Chandrashekar et al., 2024). Here we only observed a trend in decline in hepatic LRP-1 in both Tg2576 and WT mice fed with LDC-alcohol diet, with no change in hepatic APP (Figure 9). The relationship between the extent of liver injury/steatosis and alteration in hepatic LRP-1 and APP signaling has not been reported and needs further investigation, but based on our work, it can be speculated that the mild liver injury in the current study perhaps explains the absence of change in these hepatic proteins. However, there was a trend toward increased hepatic human Aβ_1-42_ in the alcohol-LDC treated Tg2576 (Figure 9), which corresponded with a significant decline in plasma human Aβ_1-42_ (Figure 5). The measurement of hepatic human Aβ_1-42_ in the alcohol-LDC fed Tg2576 mice, which largely have human Aβ in the brain, further suggests a role of the liver in removing Aβ from the periphery. Notably, the levels of human Aβ_1-42_ in the livers were 3-to-4-fold higher than mouse Aβ_1-42_ levels in the liver.

## 5 Conclusion

LDC alcohol feeding to aged Tg2576 mice for six weeks decreased survival, increased liver steatosis and endogenous mouse brain Aβ_1-42_ levels, and reduced microgliosis and spatial reference memory. Though no changes in brain human Aβ load were observed with LDC alcohol feeding, human Aβ load and microgliosis were positively correlated with serum ALT levels suggesting the modulation of the liver-brain axis by chronic alcohol-LDC feeding. Overall, these results suggest that chronic LDC alcohol intake, a model of moderate alcohol intake, produces modest but significant brain and hepatic changes that are relevant to AD pathogenesis in aged Tg2576 mice.

## Data Availability

The original contributions presented in the study are included in the article/Supplementary material, further inquiries can be directed to the corresponding author.
