# Supplementary material for: Effect of chronic alcohol feeding using the Lieber-DeCarli diet on Alzheimer’s disease pathology in Tg2576 mice
Source: Front Aging Neurosci. 2025 Mar 24;17:1526571. doi: 10.3389/fnagi.2025.1526571 (PMC11973299; doi:10.3389/fnagi.2025.1526571)
Supplement: Supplementary file 1 [file Data_Sheet_1.docx]

***Supplemental Materials***

**Supplementary Figures:**

***
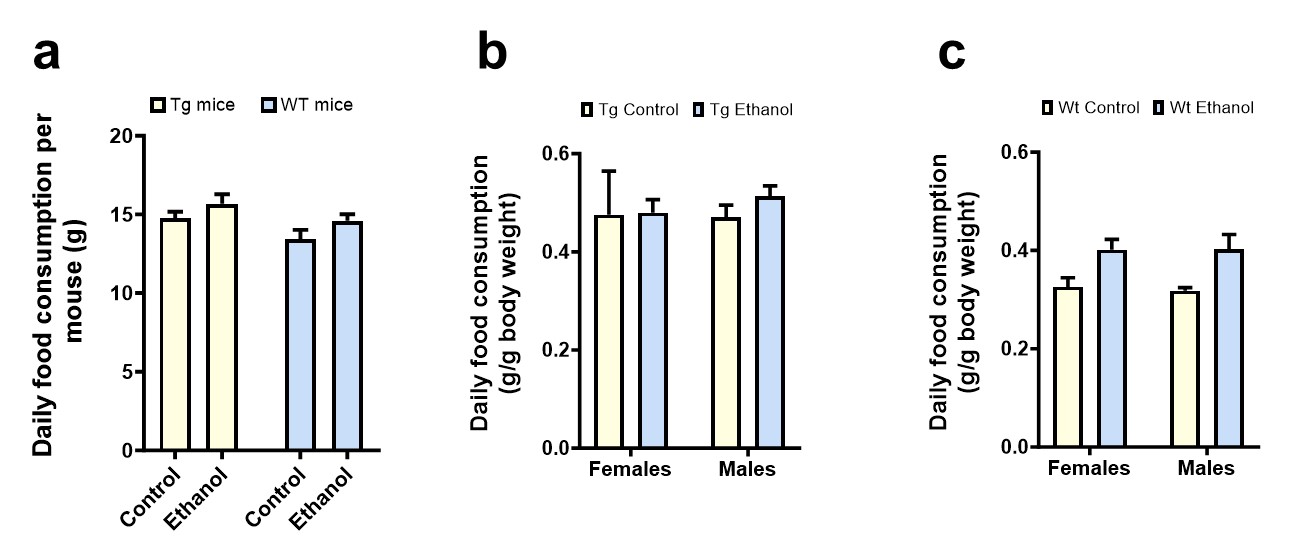
***

**Supplemental Figure 1.** The bar graph shows no changes in the average feed consumed per mouse between the control-diet-fed and alcohol-LDC-diet-fed Tg2576 mice and WT littermates (**a**). On normalizing the feed consumption by body weight, no significant effect was observed on daily food consumption in male and female Tg2576 (**b**) and WT (**c**) mice between control-diet-fed and alcohol-diet-fed mice. Data are presented as mean ± SEM of n = 7–13 per group and were analyzed using the two-way ANOVA with Holm-Sidak’s post-hoc test.





**Supplemental Figure 2.** Tg2576 mice and WT mice were combined, and the data shows a significant increase in the latency to enter the novel arm in the alcohol-LDC-diet fed compared to the control-diet-fed Tg2576 and WT mice combined. Data are presented as mean ± SEM of n = 15–18 per group and were analyzed using the two-sample t-test. *p<0.05.

**Supplemental Figure 3.** No significant changes in the brain TNF-α levels between the control- and alcohol-LDC-diet-fed Tg2576 and WT mice. TNF-α levels in TBS brain fractions, expressed in pg/mg protein, were measured by ELISA as per vendor instructions (Cat. No. 430904, Bio Legend, CA, USA). Data are presented as mean ± SEM of n = 6–13 per group and were analyzed using the two-way ANOVA.

**
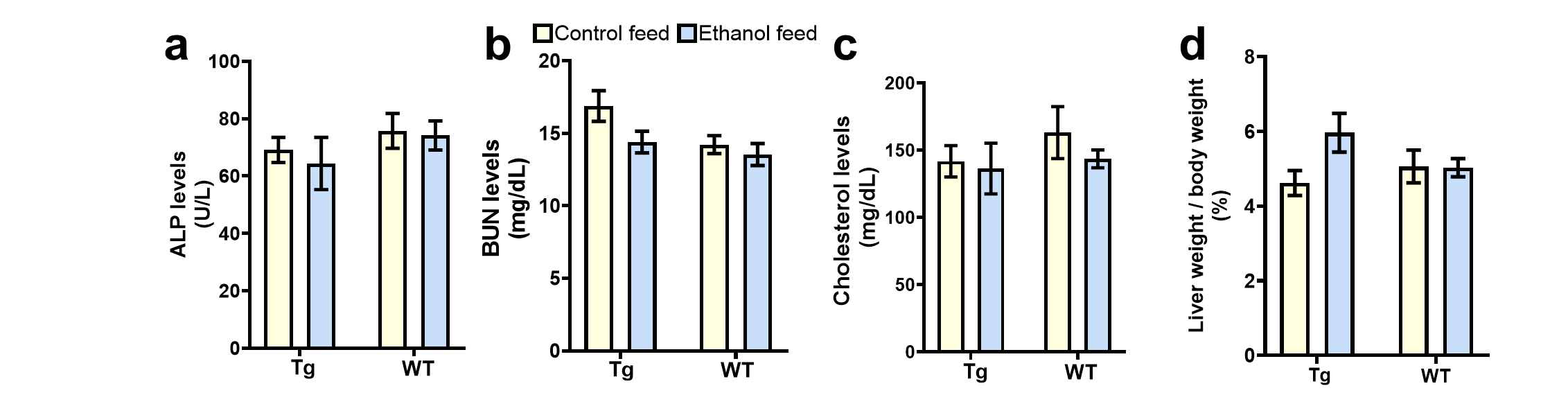
**

**Supplemental Figure 4.** Effect of alcohol-LDC-diet feeding on liver injury biomarkers (alkaline phosphatase (ALP), blood urea nitrogen (BUN), and cholesterol) in the serum samples and liver/body weight of Tg2576 and wild-type (WT) mice. The levels of ALP (**a**), BUN (**b**), cholesterol (**c**), and liver/body weight (**d**) showed no significant change in Tg2576 or WT mice fed with alcohol-LDC- or control-diet, although a strong trend toward an increase in liver/body weight was observed in alcohol-fed Tg2576 mice. Data are presented as mean ± SEM of n = 6–13 per group and were analyzed using the two-way ANOVA.


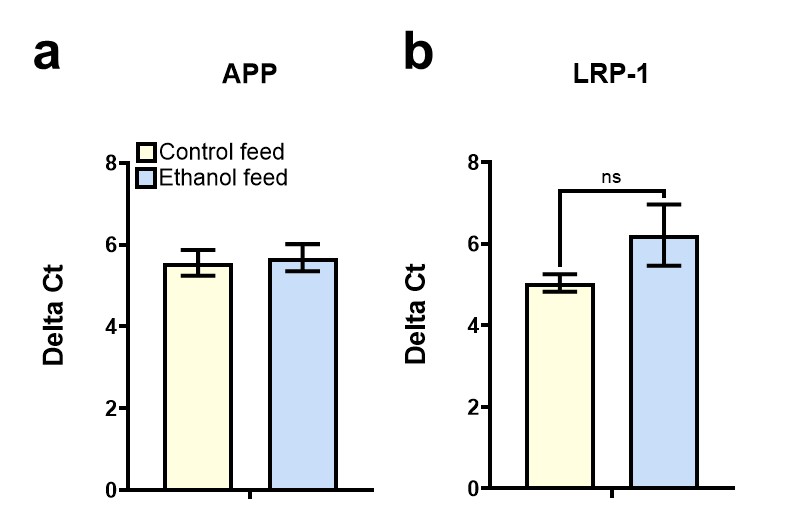


**Supplemental Figure 5.** No significant changes in the gene expression levels of mouse APP (**a**), and LRP-1 (**b**) in the liver samples of Tg2576 mice fed with alcohol-LDC- or control-diet. Data are presented as mean ± SEM of n = 4–7 per group and were analyzed using the two-sample t-test.

**
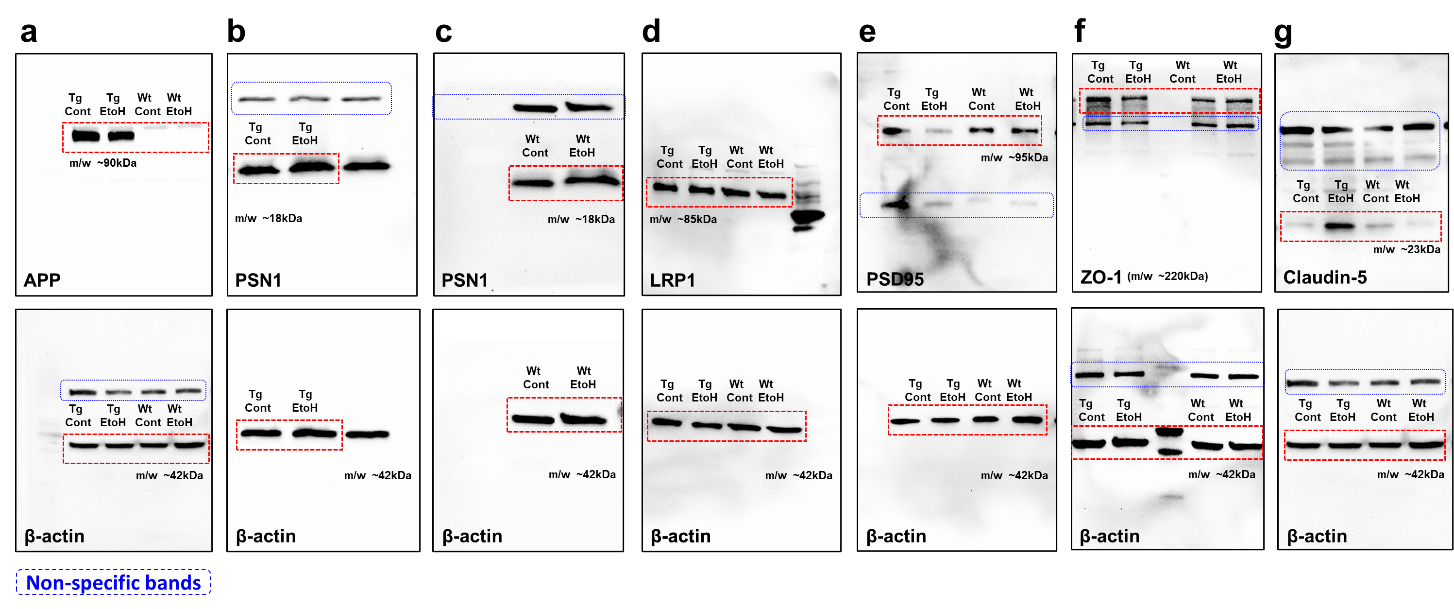
**

**Supplemental Figure 6.** Full-gel Western blot images for proteins shown in Fig. 7a-f (**a-g**). The bands shown in Fig. 7 of the article are shown in the red box above. Bands in the blue box represent non-specific bands.

**
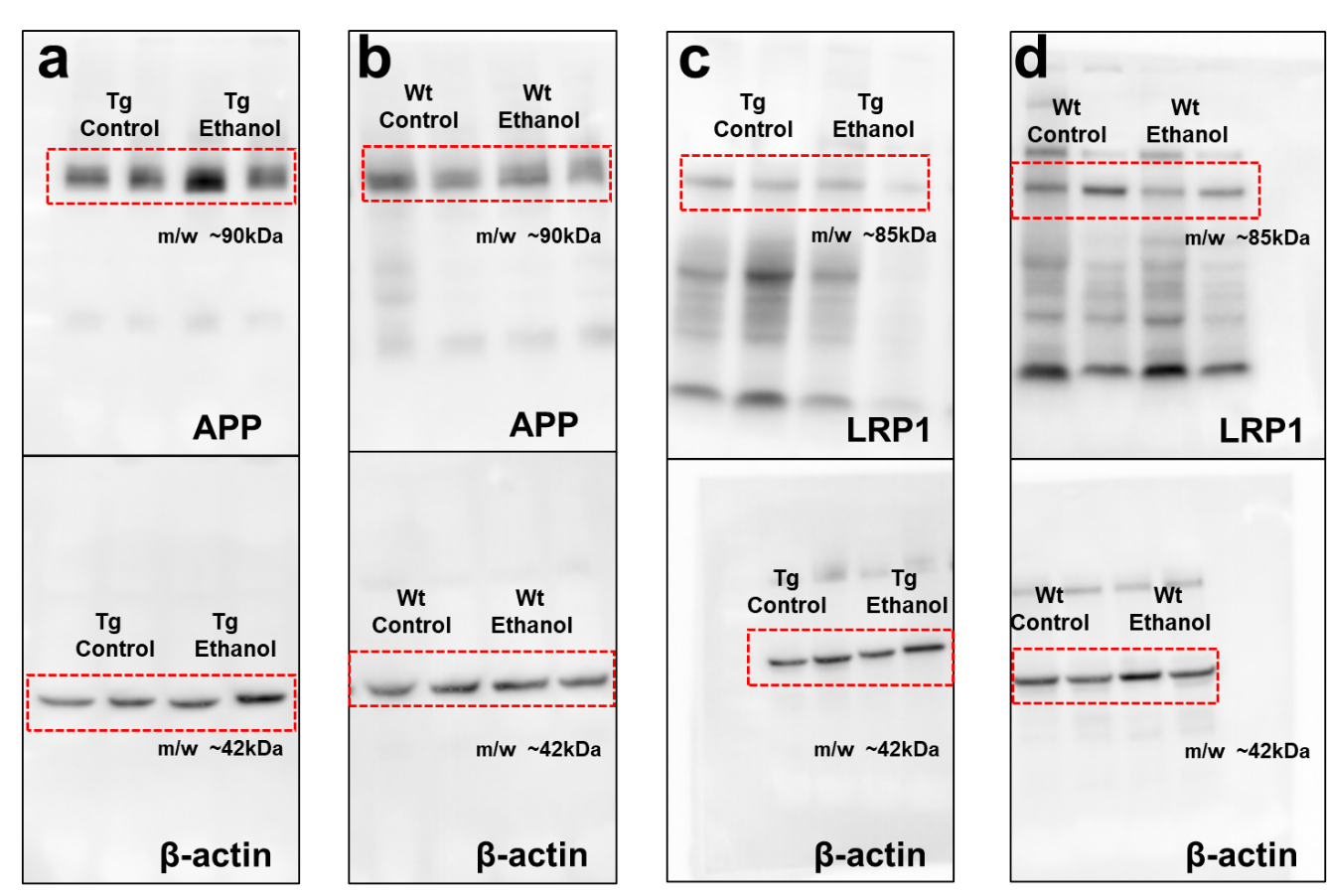
**

**Supplemental Fig. 7.** Full-gel Western blot images for proteins shown in Fig. 9c, d (**a-d**). The bands shown in Fig. 9 of the article are shown in the red box above.
